# Microcomb-driven large-scale fully connected quantum network

**DOI:** 10.1038/s41467-026-75658-6

**Published:** 2026-08-03

**Authors:** Fang-Xiang Wang, Sheng-Teng Zheng, Long Huang, Guo-Wei Zhang, Guang-Shu Wang, Wen-Jing Ding, Ze-Hao Wang, Shuang Wang, Zhen-Qiang Yin, Chang-Ling Zou, Brent E. Little, Guochao Wang, Lingxiao Zhu, Guang-Can Guo, Weiqiang Wang, Wenfu Zhang, Wei Chen, Zheng-Fu Han

**Affiliations:** 1 Laboratory of Quantum Information, University of Science and Technology of China, Hefei, China; 2 Anhui Province Key Laboratory of Quantum Network, University of Science and Technology of China, Hefei, China; 3 CAS Center for Excellence in Quantum Information and Quantum Physics, University of Science and Technology of China, Hefei, China; 4 Hefei National Laboratory, University of Science and Technology of China, Hefei, China; 5 State Key Laboratory of Ultrafast Optical Science and Technology, Xi’an Institute of Optics and Precision Mechanics, Chinese Academy of Sciences, Xi’an, China; 6 University of Chinese Academy of Sciences, Beijing, China; 7 College of Intelligence Science and Technology, National University of Defense Technology, Changsha, China; 8 School of Electronic Information and Artificial Intelligence, Shaanxi University of Science and Technology, Xi’an, China

**Keywords:** Silicon photonics, Quantum information, Solitons

## Abstract

Fully connected quantum networks enable simultaneous connection of every user to every other user and are a highly versatile and robust networking architecture. However, the scalability of such networks remains a great challenge for practical applications. Here we construct a large-scale fully connected quantum network founded on two-photon Hong-Ou-Mandel (HOM) interference, where user-to-user security is guaranteed even with an untrusted network provider. Using integrated soliton microcomb (SMC) and photonic encoding chips, we realize precise, massively parallel frequency generation and locking, high-visibility HOM interference and measurement-device-independent (MDI) quantum key distribution. The proposed architecture enables a fully connected quantum network over 200 kilometers with strict information-theoretic security via an untrusted network provider. The implemented networking architecture paves the way for large-scale fully connected MDI quantum networks across metropolitan and intercity regions.

## Abstract (web summary)

Fully connected quantum networks could enable secure communication between all users, but scalable operation with untrusted providers remains challenging. Here, the authors demonstrate a microcomb-driven measurement-device-independent quantum network using integrated photonics over 200-km fibre.

## Introduction

Quantum key distribution (QKD)^1^ monitors eavesdropping by utilizing fundamental principles of quantum mechanics and enables information-theoretically secure communication even under quantum computing attacks^2–5^. QKD has made significant achievements in recent years and has demonstrated secure key distribution capability over long distances^6–9^ and in field networks ranging from fibre to space-to-ground quantum networks^10–12^. The security of such networks depends on the trustworthiness of network providers, which weakens the quantum-guaranteed security. By leveraging two-photon Hong-Ou-Mandel (HOM) interference^13^ and post-selected Bell state measurement (BSM), measurement-device-independent QKD (MDI-QKD) closes all detection loopholes of the QKD system and thus offers a higher security level in practical implementation^14,15^. However, although MDI-QKD has achieved long-distance secure key distribution^16^, implementing a large-scale MDI-QKD network remains a formidable task, as an N-user point-to-point MDI-QKD network requires precise frequency locking between N remote laser sources and only the three-node proof-of-principle network was verified^17–20^.

A fully connected quantum network, which enables simultaneous interconnection among all terminal users, represents the most versatile and robust networking architecture^21^. However, the fully connected network is resource-consuming and suffers from an ultra-low secure key rate^21–24^. By executing the MDI-QKD protocol with two-photon HOM interference, the end-to-end secure key rate of a fully connected quantum network could be significantly improved. Critically, this improvement does not require trust in the network providers. By placing the costly receivers with single-photon detectors at the network provider, MDI-QKD enables a high-security network with low user costs. However, an N-user fully connected MDI quantum network requires precise frequency locking between ${{{\mathcal{O}}}}({N}^{2})$ laser pairs with parallel frequency channels. High-precision commercial laser sources are typically locked to specific transition lines of atomic or molecular gases and are only usable for certain wavelengths. This poses formidable technical challenges in establishing a fully connected MDI quantum network in terms of scale and cost. Although the plug-and-play MDI quantum network, which shares a common frequency source among all users, was proposed^25^ and demonstrated^26^ to circumvent the massive frequency resource preparation and cross locking, it faces critical security threats at the transmitters^27–29^, which weakens the potential security level of the MDI quantum network in practical deployments^5^. To address those critical security threats, countermeasures such as those proposed in refs. ^30,31^ might be necessary.

Recently, integrated photonic technology has shown advantages in reducing quantum system size, weight and power consumption, as well as production cost^32–36^. Moreover, integrated photonic circuits enable compact frequency multiplexing techniques to increase communication bandwidth and scale up optical networks in both classical systems^37^ and quantum systems^11^. In particular, on-chip dissipative Kerr^38–40^ solitons are promising for realizing massively parallel high-speed classical^37,41^ and quantum^42^ backbone links. A single-soliton microcomb (SMC) chip can offer hundreds of parallel frequency sources with inherent frequency and phase locking. By locking the seed (pump) laser and repetition rate, massively parallel HOM interference has been demonstrated between independent SMC chips, indicating the feasibility of realizing a large-scale fully connected MDI quantum network^43^.

Here, we demonstrate a high-performance fully connected MDI-QKD network using integrated photonic technology. Every user in the network has 200 frequency channels to connect and share information with other ones. Each user requires only a single seed laser with precise frequency locking. All seed lasers operate at the same wavelength, which perfectly solves the massive parallel frequency-locking challenge. The MDI-QKD transmitters are implemented with silicon photonic chips and operated at 2.5 GHz. Taking the finite-size effect into account, the network achieves an average secure key rate of 62 bps at 200 km for each user-to-user connection. The user scale and secure key rate of our network represent an improvement of one and three orders of magnitude, respectively, over those of previously reported fully connected quantum networks. The implemented networking architecture facilitates the practical deployment of large-scale fully connected quantum networks with a high level of security.

## Results

**Fig. 1:**
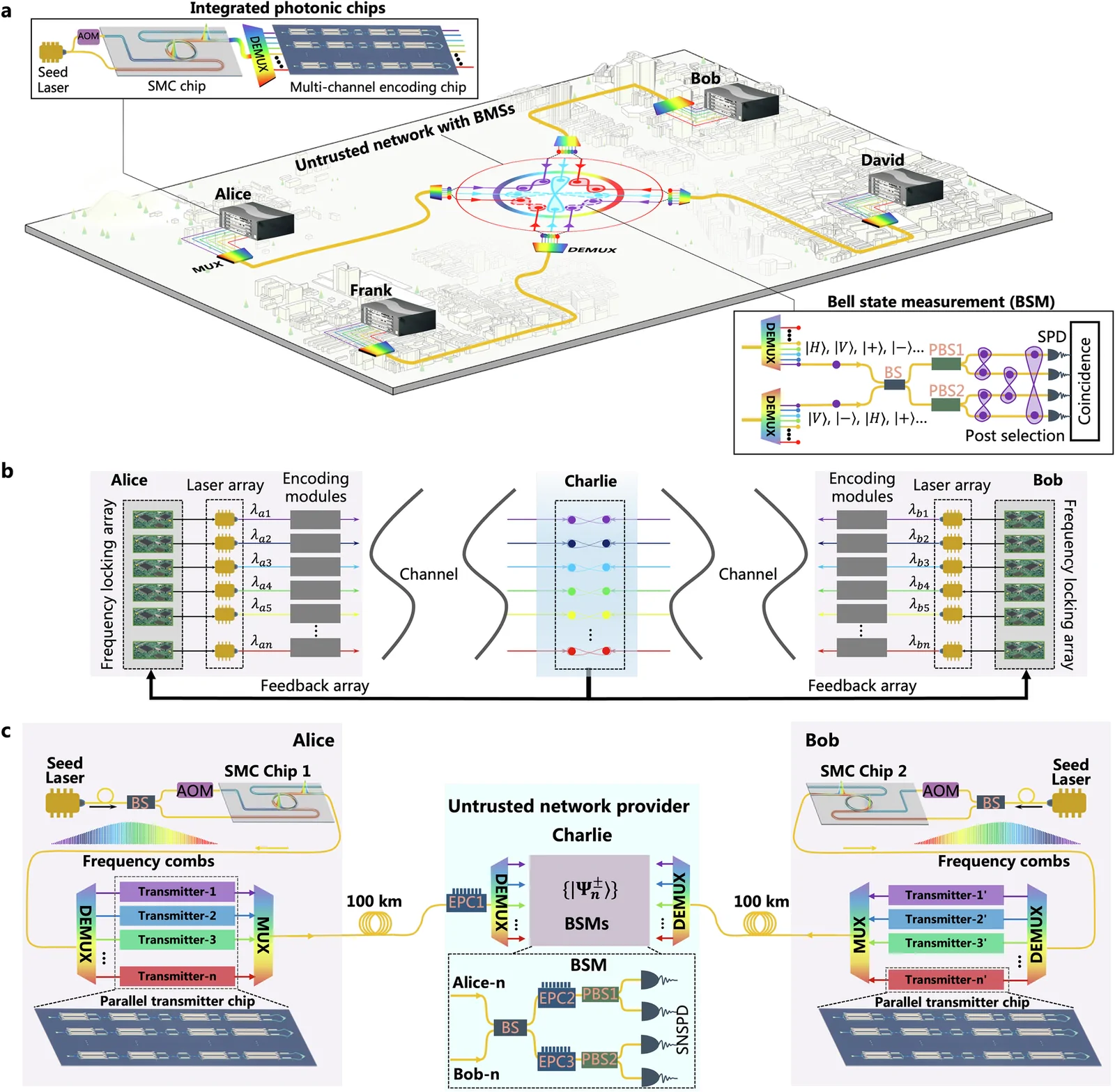
Fully connected quantum network with untrusted network provider. **a** Schematic of a fully connected MDI-QKD network (four users as an example), in which all terminal users can be connected to each other simultaneously without trusting the network provider and each user requires only a frequency-locked seed laser source. **b** The typical frequency-locking laser arrays for massively parallel MDI-QKD network, which is a formidable technical challenge. **c** Experimental setup of the subsystem of the massively parallel fully connected MDI-QKD network using SMC and QKD transmitter chips over 200 km.

A massively parallel fully connected MDI-QKD network is constructed using SMC and MDI-QKD transmitter chips, as shown in Fig. 1a. Frequency-locked SMCs are employed as multi-channel carriers and quantum states are prepared with multi-channel encoding transmitter chips at all QKD network terminals. Then quantum states over hundreds of channels are sent to the untrusted quantum network. The network distributes these channels to different connection links and executes the post-selected Bell state measurements (BSMs). All users realize secure information sharing simultaneously after the network provider publicly announces the BSM results. According to the fundamental principles of quantum mechanics, the quantum relay in the network cannot obtain any information shared between end users during Bell state measurements^14,15^. We utilize SMC and transmitter chips to implement the massively parallel MDI-QKD, where the seed laser frequency and repetition rate are locked to align all the soliton comb lines. The frequency locking of SMC chips is fulfilled locally and demands no remote operation from the network provider or other end users. The independent frequency-locked SMC chips can provide hundreds of available pairs of frequency comb teeth with high HOM interference visibilities for fully connected MDI-QKD networks. Hence, the proposed architecture here significantly simplifies the implementation complexity and expands the user scale of a fully connected quantum network, while the typical frequency locking method for ${{{\mathcal{O}}}}({N}^{2})$ laser pairs with different wavelengths faces enormous technical challenges (as shown in Fig. 1b) and only a three-node network was realized^17,44^. Figure 1c shows the sub-system of the massively parallel fully connected MDI-QKD network based on independent SMC and transmitter chips over 200 km. At the end user side (Alice and Bob), part of the seed laser acts as the pump laser and is coupled into the micro-ring resonator (MRR) on the SMC chip to generate a wideband frequency comb source. The remaining part of the seed laser is frequency-shifted by an acousto-optical modulator (AOM) and acts as an auxiliary laser to balance the thermal effects inside the MRR. The spectrum range of a single SMC chip is over 100 nm, which can provide more than 200 frequency channels with high signal-to-noise ratios (SNR, >20 dB) and a large frequency interval (~49 GHz). The frequency comb teeth are then demultiplexed by an arrayed-waveguide grating (AWG) filter and distributed to the transmitter chip. The transmitter chip chops the frequency teeth into narrow pulses, then attenuates and encodes the pulses into single-photon-level polarization states. The two bases of polarization states are $Z:=\{\left\vert H\right\rangle,\left\vert V\right\rangle \}$ and $X:=\{\left\vert+\right\rangle=\frac{1}{\sqrt{2}}(\left\vert H\right\rangle+\left\vert V\right\rangle ),\left\vert -\right\rangle=\frac{1}{\sqrt{2}}(\left\vert H\right\rangle -\left\vert V\right\rangle )\}$, where $\left\vert H\right\rangle$ ($\left\vert V\right\rangle$) denotes the horizontal (vertical) polarization. The encoded pulsed photons are then transmitted to the untrusted quantum network provider Charlie over a 100-km fibre channel, corresponding to a user-to-user distance of 200 km. The polarization drifts of fibre channels are compensated using electrically driven polarization controllers (EPC). Charlie demultiplexes the received photon pulses and performs post-selected BSM detection with two-photon coincidences. The BSM post-selects only $\left\vert {{{\Psi }}}^{\pm }\right\rangle=\frac{1}{\sqrt{2}}\left\vert HV\right\rangle \pm \left\vert VH\right\rangle$ and consists of a non-polarizing beam splitter (BS, for HOM interference) and two polarizing beam splitters (PBS, for polarization measurement). Charlie announces all two-photon coincident measurement results publicly. Alice and Bob then successfully share a string of secure keys by executing a post-processing procedure according to Charlie’s announcements. The security of the key sharing process is independent of Charlie’s loyalty. Hence, by flexibly allocating frequency channels, the proposed architecture can be used to realize both massively parallel high-speed backbone links and fully connected quantum networks with untrusted quantum network providers.

### Local locking of independent SMC chips

**Fig. 2:**
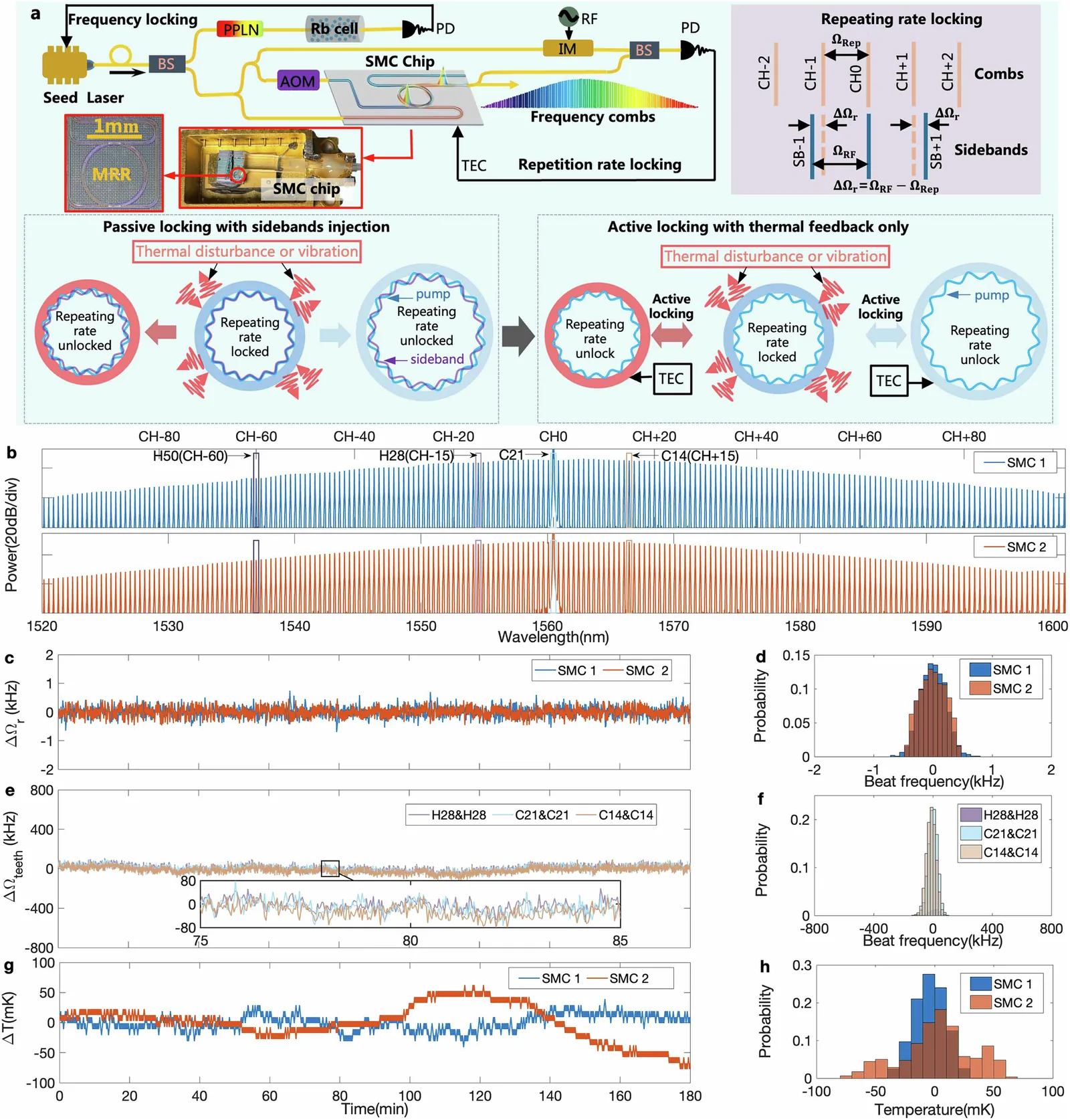
Locally fully stabilized SMC chips. **a** Schematic diagram of the SMC local frequency locking setup. The seed laser is locked to a local rubidium cell. The repetition rate of the SMC chip is actively locked to a 49-GHz local radio frequency (RF) reference via thermal feedback. IM: intensity modulator. **b** Optical frequency spectra of two independently locked SMC chips. **c**, **d** The concurrent repetition rate fluctuations in real time and statistics of two independent SMCs over 3-h running. **e** The real-time beat frequencies of three pairs of comb teeth within the concurrent time span, and **f** their corresponding statistical distributions. **g**, **h** The monitored real-time operating temperature fluctuation and corresponding statistical distributions of the independently repetition-rate-locked SMC chips.

The photon frequency alignment at different transmitters is a fundamental requirement for MDI-QKD, which means that all the independent SMC chips in the network should be locked to the same reference. Figure 2a presents the local frequency locking scheme of the SMC chip in the proposed fully connected MDI-QKD network. The two degrees of freedom of the on-chip soliton microcomb are the seed laser frequency and repetition rate, which, respectively, determine the center frequency (CH0) and repetition rate Ω_*Rep*_ of the frequency comb. The parallel seed lasers are locally locked to the D2 transition line of rubidium cells (^87^**Rb**, 5^2^S_1/2_ → 5^2^P_3/2_). To lock the repetition rate Ω_*Rep*_ of the SMC, the seed laser is modulated by a radio-frequency (RF) reference Ω_*RF*_ via an intensity modulator (IM). Then the beat note between the first-order sidebands of the SMC and the RF-modulated seed laser is detected using a photodetector (PD) with a 1-GHz bandwidth. Benefiting from the PD bandwidth limitation, only the difference frequency ΔΩ_*r*_ = Ω_*RF*_ − Ω_*Rep*_ is passively filtered out. This signal is then used as the error signal to lock Ω_*Rep*_ by feeding back the operating temperature of the MRR. The repetition rate is actively stabilized to the frequency of the local RF reference by feeding back the thermal effect of the SMC chip to ensure ΔΩ_*r*_ approaching to zero. Compared to the intracavity-sideband-locking scheme^43^, the proposed active locking method avoids the unstable competitive mechanism, thus, expands the locking range and enhances the long-term stability against thermal disturbance and vibration (Fig. 2a).

Figure 2b shows the optical spectra of two independent SMC chips, which could provide more than 200 comb teeth, each with an SNR exceeding 20 dB. The stability of the repetition rates (ΔΩ_*r*_) of both SMC chips is shown in Fig. 2c, d. The peak-to-peak and standard deviation of the repetition rate fluctuations are only 1442 Hz and 215 Hz, respectively, over 3 h. Figure 2e, f shows the beat frequencies ΔΩ_*teeth*_ and their statistical distributions for three pairs of comb teeth (CH0&CH0, CH+15&CH+15, and CH-15&CH-15, corresponding to C21&C21, C14&C14 and H28&H28 of the standard ITU channels, respectively) over the concurrent time period. The corresponding standard deviations of the beat frequencies are 30.7 kHz, 31.1 kHz and 29.6 kHz, respectively, which are limited by the characteristic linewidth of the ^87^**Rb** atomic transition process^45^. The enlarged view in Fig. 2e shows no statistically significant difference among these tooth pairs. The high degree of homogeneity among the observed beat frequencies provides compelling evidence for the consistency of the repetition rates between these independent SMC chips.

The peak-to-peak thermal-tuning range is less than 150 mK over 3 h (Fig. 2g, h) under active repetition-rate stabilization. It is significantly smaller than the SMC survival temperature range of 2K^43^. Compared to microwave injection locking, active temperature feedback can effectively reduce the intracavity power, mitigating the influence of background waves generated by injected sidebands on soliton formation. Additionally, active locking offers stronger resistance to environmental perturbations^46^.

Local frequency locking to the same but independent references significantly simplifies network complexity, making large-scale networks more robust for practical deployment. The long-term frequency-locking precision and stability of comb teeth, enabled by locally fully locked SMCs, satisfy the stringent requirements for a high-performance, large-scale, massively parallel MDI-QKD network.^34^.

### Transmitter chip characterization and HOM interference

**Fig. 3:**
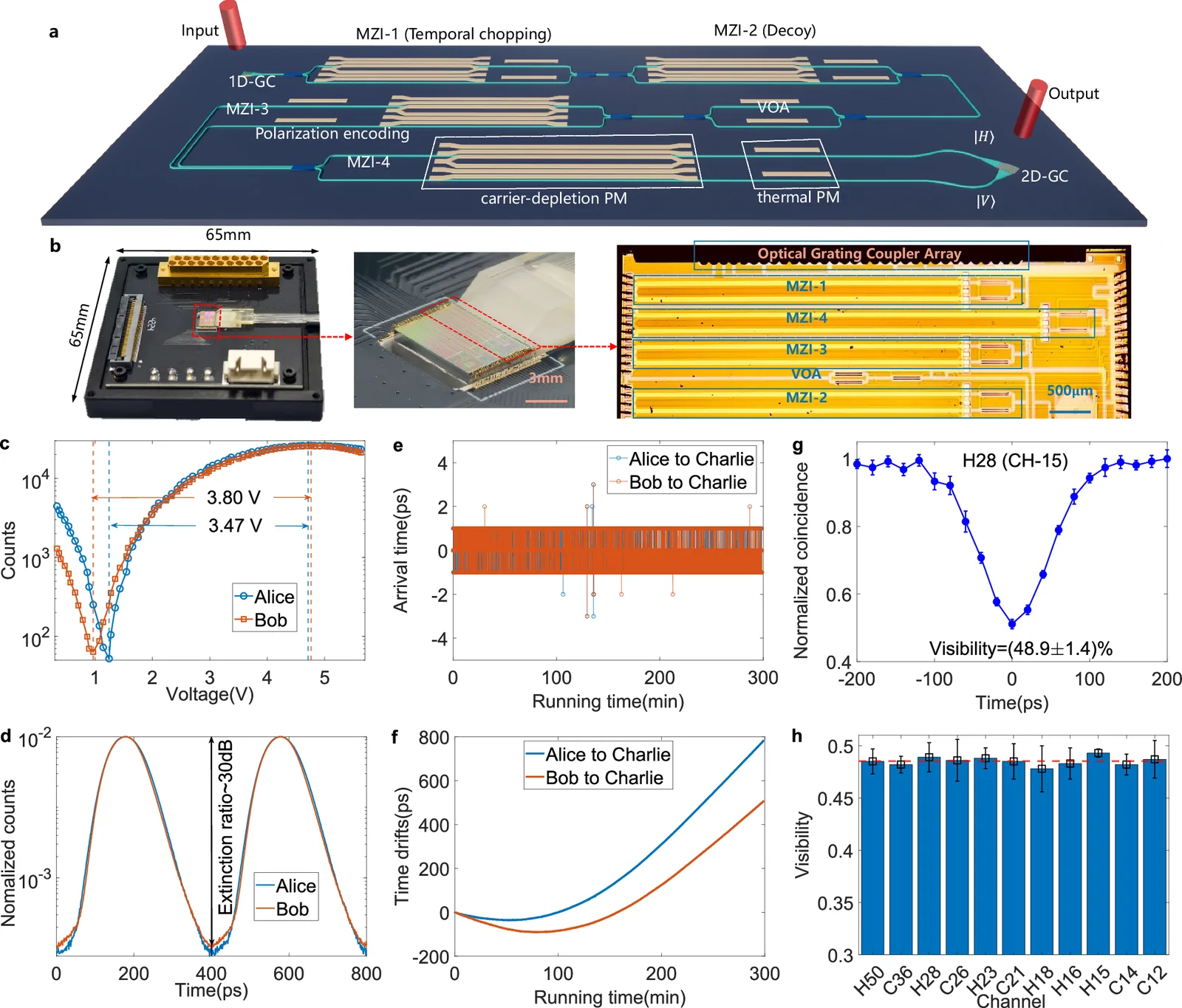
Transmitter chip characterization. **a** Schematic of the polarization-encoded transmitter chip (4 MZIs + VOA). **b** The images of the optoelectronically packaged transmitter chip. **c** Voltage-dependent transmittance of the MZIs of both transmitter chips. **d** The normalized temporal intensity profiles of the pulsed comb teeth modulated by the on-chip MZI, which includes the time jitter of the SNSPD. Arrival time drifts of photon pulses **e** with, and **f** without time-drift compensation after transmission over a 100-km fibre channel. **g** HOM interference fringe of the 90-ps tooth pair H28& H28. **h** HOM visibilities of tooth pairs from H50& H50 to C12& C12 (corresponding to CH-60& CH-60 to CH+19& CH+19 of the comb teeth).

High-performance integrated transmitter chips are indispensable for realizing a low-cost, large-scale, fully connected quantum network. The silicon-photonic-chip-based MDI-QKD transmitters operate at 2.5 GHz. As shown in Fig. 3a, the transmitter chip incorporates four electro-optic and one thermo-optic Mach-Zehnder interferometers (MZI) in a monolithic design. Both carrier-depletion and thermal-tuning phase modulators (PM) are employed for the electro-optic MZIs. The optical carrier is coupled into and out of the transmitter chip through 1D and 2D grating couplers (1D-GC and 2D-GC), respectively. MZI-1 to MZI-4 operate in push-pull modulation mode, where MZI-1 and MZI-2 chop the CW optical carrier into pulsed signals and realize decoy-state intensity modulation. The subsequent thermo-optic MZI serves as a variable optical attenuator (VOA) to achieve single-photon-level output. MZI-3 and MZI-4 jointly prepare the mutually unbiased polarization bases *Z* and *X*. The upper (lower) path of MZI-3 is mapped to $\left\vert H\right\rangle$ ($\left\vert V\right\rangle$) via the 2D-GC. High-speed switching between $\left\vert H\right\rangle$ and $\left\vert V\right\rangle$ is achieved through carrier-depletion PMs in MZI-3. The X basis is realized by modulating the carrier-depletion PMs in both MZI-3 and MZI-4. Figure 3b illustrates the compact optoelectronic packaging (65 × 65 × 16 mm^3^) of the transmitter chip, with magnified views showing the photonic integration details. Figure 3c displays the voltage-dependent modulation characteristics of the MZIs at a repetition rate of 2.5 GHz, where the half-wave voltages are 3.47 V for Alice and 3.80 V for Bob. As presented in Fig. 3d, the optical carriers are chopped into 95-ps pulses (FWHM) through MZI-1, with a 30-dB extinction ratio for both chips. The propagation of narrow photon pulses through the long-distance fibre channel is subject to dynamic temporal shifts, which impair two-photon interference visibility. The photon arrival time is monitored and compensated in real time. This real-time compensation effectively suppresses the arrival time drifts from Alice to Charlie and Bob to Charlie to ~2 ps (Fig. 3e), which is two orders of magnitude smaller than those without compensation (Fig. 3f).

Based on the high-performance SMC and transmitter chips, high-visibility HOM interferences between Alice and Bob are realized using the 95-ps photon pulses, where a multi-channel superconducting single-photon detector (SNSPD) is used and the count rate per channel is about 1 Mcps. Figure 3g shows the HOM interference dip of H28&H28. The HOM interference visibility is (48.9 ± 1.4)%, approaching the theoretical limit of 50.0%. Figure 3h displays HOM interference visibilities for tooth pairs spanning H50&H50 to C12&C12. All tooth pairs covering 80 channels exhibit consistent interference quality, and the average visibility is 48.5% (red dashed line). See Supplementary Note 6 for detailed HOM interferences of these channels.

### Secure key sharing rate

Using the highly stable SMCs and high-SNR encoding transmitter chips, we demonstrate massively parallel MDI-QKD over a 200-km fibre channel with high secure key rates, requiring no trusted quantum network provider. The polarization-based MDI-QKD system adopts the four-intensity decoy-state method. The high frequency-uniformity of the SMCs and the 30-dB extinction-ratio transmitter chips ensure low QBERs in quantum states preparation. Although polarization encoding offers the advantage of a high secure key rate, polarization-based MDI-QKD^34,47^ had not addressed the rigorous polarization compensation issue and its practicality had yet to be proven. Here, we develop a two-stage polarization compensation strategy to simultaneously compensate the mutually unbiased bases (both Z and X bases) over distant fibre channels. The stochastic parallel gradient descent (SPGD) algorithm^48^ together with EPCs, is used to dynamically compensate the polarization drifts over the 200-km fibre channels (see Supplementary Note 3 for details).

**Fig. 4:**
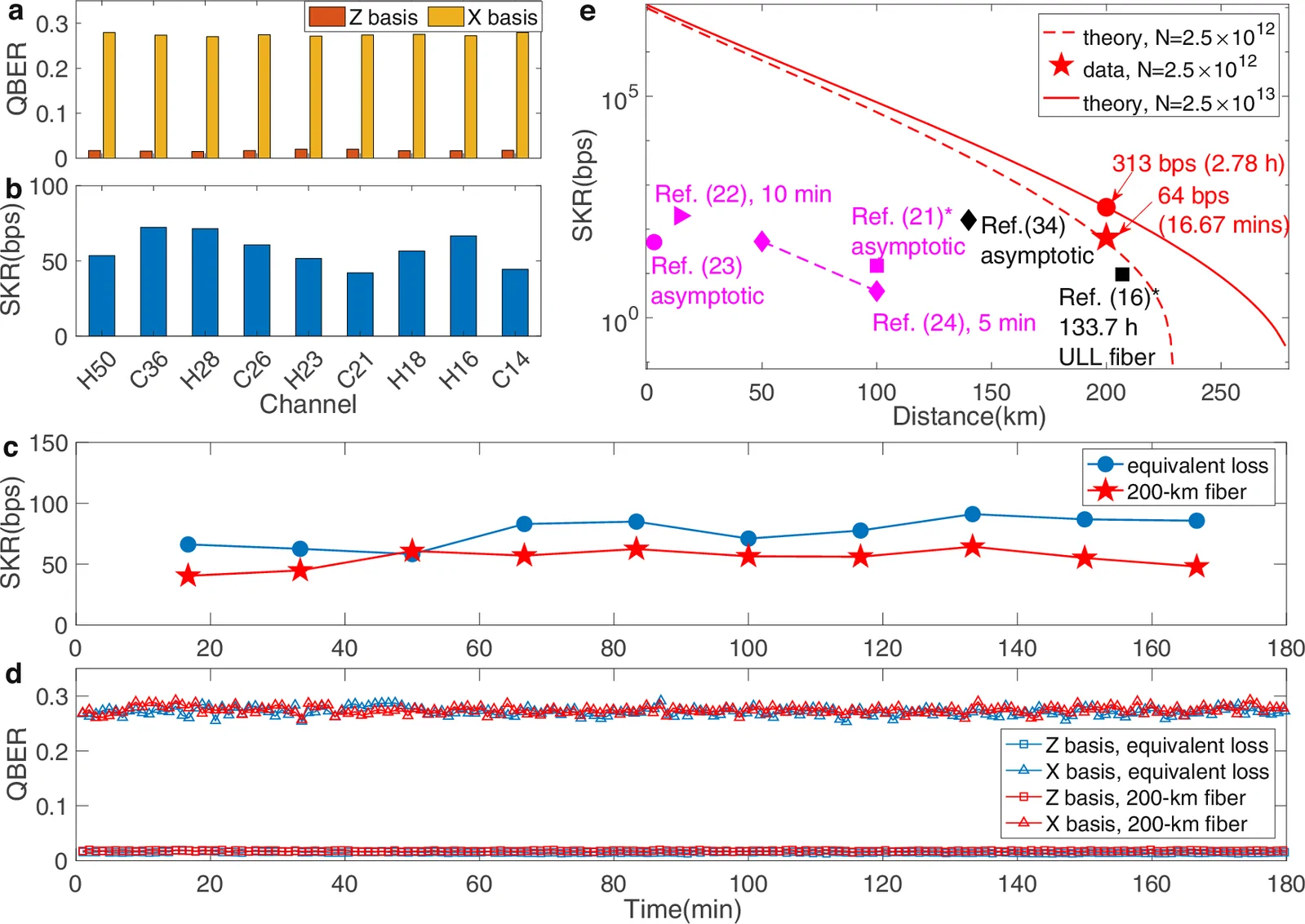
Secure key rates and QBERs of the integrated parallel MDI-QKD system over a 200 km fibre. **a** QBERs and **b** secure key rates of different tooth pairs spanning H50& H50 to C14& C14. **c**, **d** Real-time secure key rates and QBERs of H28& H28 over 3 h running, where secure keys are extracted every 1000 s. The blue and red colors denote the equivalent-loss and 200-km fibre channels, respectively. **e** Secure key rates per channel versus distance. ^*^Reference ^16^ used ultra-low-loss (ULL, 0.16 dB/km) fibre and ref. ^21^ simulated the channel with attenuators.

Figure 4a, b shows the QBERs and secure key rates of different MDI-QKD channels using comb tooth pairs spanning H50&H50 to C14&C14 (covering 76 channels). The secure key rate is calculated by taking into account the finite-size effect^49^, where the failure probability is kept below 1 × 10^−10^ and the accumulation time for each key extraction round is 1000 s (~16.67 minutes). The average QBER and secure key rate of the *Z* (*X*) basis across all channels are (1.7 ± 0.2)% ((27.4 ± 0.3)%) and 58 bps, respectively, for a finite number of sent pulses accumulated over 1000 s (~16.67 minutes). See Supplementary Note 2 for a detailed discussion of the decoy state parameters and finite-size effect analysis. Figure 4c, d shows the long-term running secure key rates and QBERs. The system performance remains consistent with that observed in the initial 1000 s, demonstrating the effectiveness of the dynamic polarization compensation. The secure key rate and QBERs remain stable for both equivalent-loss and 200-km standard single-mode fibre channels. The system attains a secure key rate of up to 64 bps over a 200-km fibre channel. The consistency of performance between the equivalent-loss and 200-km-fibre channels demonstrates the high stability of the system for a long-distance, large-scale, fully connected quantum network with untrusted network providers. Figure 4e compares the secure key rates of the proposed fully connected quantum network with previous advances. Notably, the per-channel key rate here is substantially higher, while the data accumulation time required to extract a finite-size key block is much shorter than the 10–133 h needed by previous single-channel discrete-device^16,50^ and chip-based MDI-QKD systems^34^. By increasing the accumulation time to 10,000 s (~2.78 h), the secure key rate per channel reaches 313 bps at 200 km (red solid line), which is two orders of magnitude higher than the previous record achieved with a 200-km ultra-low-loss (ULL) fibre^16^. Furthermore, compared with previous fully connected quantum networks that lacked rigorous finite-size effect analysis^21–24^, the secure key rate and distance achieved by the proposed architecture are three to four orders of magnitude higher and 100 km longer, respectively.

## Discussion

We present a fully connected quantum network architecture that simultaneously achieves high-speed performance and large-scale deployment through high-precision manipulation of SMCs and low-QBER quantum state preparation. The integration of both optical SMCs and QKD transmitters in compact optoelectronic packaging enables a substantial reduction in infrastructure expenditure, while maintaining network-provider-independent, quantum-grade security across all network links, representing a significant milestone in the practical implementation of quantum communication networks.

The operation channels from H50 to C14 are experimentally verified for a massively parallel, fully connected MDI-QKD network. The number of channels is currently limited only by the passband range of the AWGs used in our experiments. Given the performance homogeneity of the frequency locked SMC chips and the ultra-low crosstalk among these massive comb teeth (see Supplementary Note 5 for details), the proposed architecture can support a fully connected quantum network with thousands of users when combined with both frequency multiplexing (~200 channels) and time-division multiplexing techniques. This is two orders of magnitude larger than previous implementations.

Our work realizes long-distance, polarization-encoded MDI-QKD with finite-size secure keys, in contrast to prior studies that lacked real-time polarization compensation and only evaluated asymptotic secure key rates over long-distance fibre^34,47^. By developing a dynamic polarization compensation strategy, we achieve a mean secure key rate of 64 bps per channel (extracted every 16.67 min) over a 200-km fibre for long-term operation. This significantly benefits real-time root key update, whereas the key update intervals in previous MDI-QKD demonstrations were 10–133 h^16,34^. This removes a major obstacle to establishing an intercity fully connected MDI-QKD network. It is worth noting that the secure key sharing rate per pulse of our system is also much higher than previous works^16,17,50^ and demonstrates a significant advancement in high-speed MDI-QKD systems. Since the locking uncertainties of the pump laser and the repetition rate of the SMC are only 31 kHz and 215 Hz, respectively, our SMC source meets the frequency stability requirement of mode-pairing protocols and can further increase the secure key rate^51,52^. Thanks to the high-performance transmitter chips, our system shows favorable results when considering potential side-channel security loopholes (see Supplementary Note 8 for details)^53,54^.

Furthermore, the proposed network architecture can also be simply modified to a quantum-repeater-based quantum network by taking the coherent comb teeth as pump lasers to generate entangled photon pairs and realizing entanglement swapping with untrusted quantum relays. Such a network can support the same performance for each user-to-user link, as the source brightness of each user is identical and independent of the network scale. Therefore, this architecture paves the way for the deployment of high-speed, large-scale, fully connected quantum networks involving untrusted network providers across metropolitan and intercity regions. It not only enhances the practicality of deployment but also delivers an alternative design perspective for secure fully connected quantum networks.

## Methods

### SMC and transmitter chips

The SMC chips are encapsulated using a butterfly-shaped optoelectronic packaging technique. The butterfly package contains the MRR chip coupled with a polarization-maintaining fibre array, a thermistor, and a thermoelectric cooler mounted beneath the chip.

The transmitter chip was fabricated using a commercial 130 nm active silicon-on-insulator (SOI) platform. It is based on a 200 mm SOI substrate with a 220 nm top silicon layer and a 2 μm buried silica oxide layer. The light is coupled into and out of the chip via grating couplers with a 70 nm etch depth and is transmitted through a single-mode strip waveguide designed for 1550 nm with a width of 450 nm. The thermo-optic phase shifters are implemented using titanium nitride (TiN) metal heaters, while high-speed electro-optic phase shifters employ carrier depletion via a four-level lateral implantation junction. The full chip measures 6.3 mm × 4.7 mm (including other structures), while the MDI-QKD transmitter section occupies only 6.3 mm × 2.2 mm. For optoelectronic packaging, the chip was coupled to a fibre array and wire-bonded to a printed circuit board (PCB). Thermal stabilization was achieved through a precision control unit comprising a heat sink, thermistor, and thermoelectric cooler (TEC), maintaining temperature stability within 0.01 K. The complete packaged module, which integrates modulators and MZIs supporting two parallel encoders, measures 65 mm × 65 mm × 16 mm, with the size primarily constrained by the multi-channel electrical interfaces.

### MDI-QKD and secure key rate

The MDI-QKD protocol adopts two mutually unbiased bases (MUBs) and a four-intensity decoy state method. The secure key rate is calculated by taking into account the finite-size effect:

1 $$R={p}_{{s}_{A}}{p}_{{s}_{B}}\{{a}_{1}^{s}{b}_{1}^{s}{y}_{11,Z}^{L}[1-h({e}_{11}^{p,U})]-{f}_{e}{Q}_{ss}h({E}_{ss})\}-\frac{1}{N} \\ \left({\log }_{2}\frac{8}{{\varepsilon }_{cor}}+2{\log }_{2}\frac{2}{\varepsilon ^{\prime} \hat{\varepsilon }}+2{\log }_{2}\frac{1}{2{\varepsilon }_{PA}}\right)$$

where ${p}_{{s}_{A}}$ and ${a}_{1}^{s}$ (${p}_{{s}_{B}}$ and ${b}_{1}^{s}$) are the sending probability and the single-photon number of the signal pulses of Alice (Bob), respectively; ${y}_{11,Z}^{L}$ and ${e}_{11}^{p,U}$ are, respectively, the single-photon pair yield and phase-flip error rate of the single-photon pairs in the *Z* basis; *Q*_*ss*_ and *E*_*ss*_ are the gain and QBER of the pulse pairs in the *Z* basis, respectively; $h(x)=-x{\log }_{2}x-(1-x){\log }_{2}(1-x)$ is the Shannon entropy; *f*_*e*_ is the efficiency factor of the error correction method; *ε*_*cor*_ and *ε*_*PA*_ are the failure probabilities of error correction and privacy amplification, respectively; $\varepsilon {\prime}$ and $\hat{\varepsilon }$ are the coefficients used in the chain rules of smooth min- and max-entropy.

The photon pulse intensities are defined as signal (*s*), decoy (*μ*, *ν*), and vacuum (*ω*) states. Alice and Bob possess symmetric channels and share the same decoy parameters. The *Z* basis only prepares the *s* state to generate secure keys, and the *X* basis prepares *μ*, *ν* and *ω* for decoy state estimation. The decoy parameters are optimized to maximize the secure key rate. In the experiments, the sending intensities {*s*, *μ*, *ν*} and probabilities {*p*_*s*_, *p*_*μ*_, *p*_*ν*_} were originally set to {0.209, 0.170, 0.056} and {0.453, 0.123, 0.380}, respectively. The parameters may be slightly modified for different comb-tooth channels. The error correction efficiency is *f*_*e*_ = 1.16. The sample size for the finite-size effect is *N* = 2.5 × 10^12^ (1000 s). The total failure probability is limited to <1 × 10^−10^ for both theoretical and experimental results of our work (Fig. 4). The total detection efficiency (including insertion loss) and dark count rate of the receiver (Charlie) are, respectively, 52% and 75 Hz. The FWHM of the time jitter of the SNSPD is 50 ps at a count rate of 1 Mcps.

## Supplementary information


Supplementary Information
Transparent Peer Review file


## Data Availability

All the data supporting this study are available in the paper and Supplementary Information.
