# Supplementary material for: Microcomb-driven large-scale fully connected quantum network
Source: Nat Commun. 2026 Aug 3;17:9319. doi: 10.1038/s41467-026-75658-6 (PMC13530157; doi:10.1038/s41467-026-75658-6)
Supplement: Supplementary file 1 [file 41467_2026_75658_MOESM1_ESM.pdf]

Supplementary Materials for Microcomb-driven Large-scale Fully Connected Quantum Network

Fang-Xiang Wang†, Sheng-Teng Zheng†, Long Huang†, Guo-Wei Zhang†, Guang-Shu Wang, Wen-Jing Ding, Ze-Hao Wang, Shuang Wang, Zhen-Qiang Yin, Chang-Lin Zou, Brent E. Little, Guochao Wang, Lingxiao Zhu, Guang-Can Guo, Weiqiang Wang*, Wenfu Zhang*, Wei Chen*, Zheng-Fu Han

†These authors contributed equally to this work.

*Corresponding authors: W. W. (wangwqopt@163.com), W. Z. (wfuzhang@opt.ac.cn), W. C. (weich@ustc.edu.cn).

Supplementary Note 1 – The local locking and scheme and encoding method

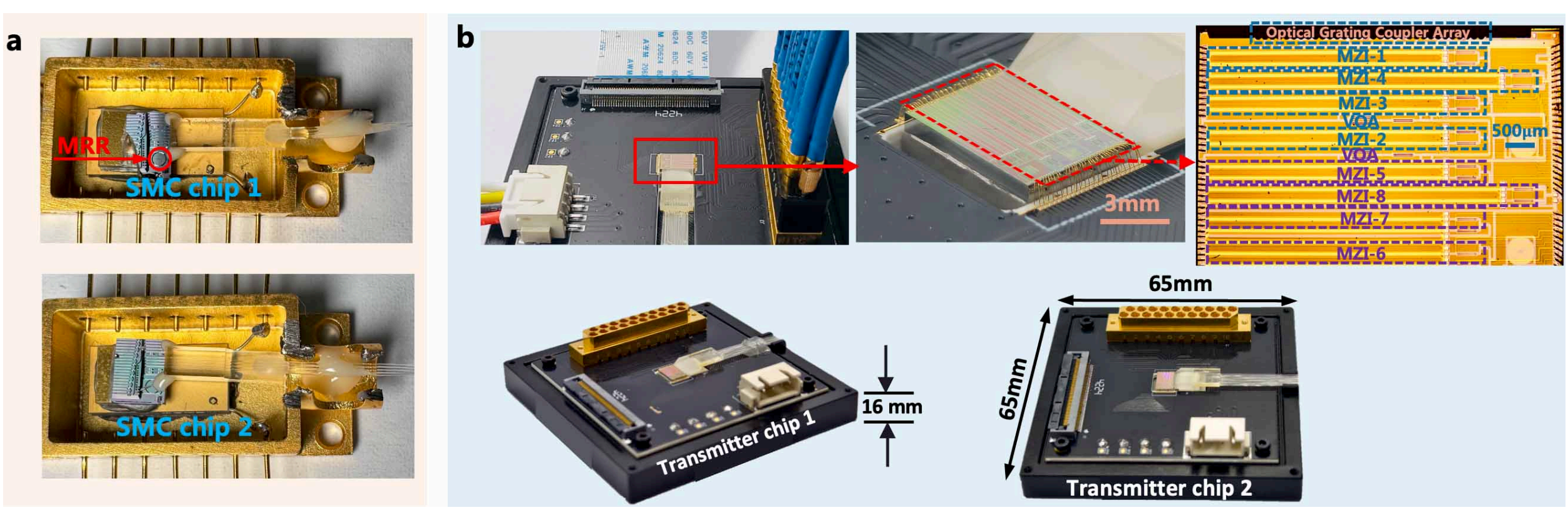

Supplementary Figure 1: **Photographs of SMC and transmitter chips. a**, SMC chips and **b**, MDI-QKD transmitter chips with optoelectronic package.

The photograph of soliton microcomb (SMC) chips is shown in Supplementary Fig. 1a. Part of the narrow-linewidth seed laser (1560.48 nm) is first doubled by a periodically poled lithium niobate (PPLN) waveguide and locked to the D^2 line of ^{87}**Rb** ($5^2S_{1/2} \rightarrow 5^2P_{3/2}$) by the modulation transfer spectroscopy technique. The remaining locked seed laser is split into two paths. The first path is amplified by a commercial EDFA and coupled into the MRR directly as the pump laser. The second path is frequency shifted ∼150 MHz by an acousto-optical modulator (AOM) and then amplified and coupled into the MRR as the auxiliary laser to balance the thermal effects inside the

MRR. By carefully adjusting the temperature of the SMC chip, the pump and auxiliary lasers are tuned into the red- and blue-detuned regions of the MRR resonance peak, respectively, leading to the generation of the single-soliton microcomb (SMC).

Due to the frequency drift of the seed laser and ambient fluctuations such as vibrations and current noise, the repetition rate of an SMC source drifts dynamically. As given in Supplementary Fig. 2a, the peak-to-peak drift of the repetition rate approaches 100 kHz within 30 minutes. For a passive locking method with intracavity sidebands, the locking range is only about 30 kHz and lacks of long-term stability, as an MDI-QKD requires data acquisition for at least tens of minutes per key extraction round to guarantee practical security (composable security with finite-size effect). In this work, we propose an active thermal locking scheme and remove the injected sideband to avoid a competitive mechanism. This significantly expands the lock-in range and improves the long-term stability of the SMC source.

The repetition rate of the SMC is stabilized using an extracavity sideband reference. Specifically, a 49-GHz RF reference drives the intensity modulator (IM) to generate the reference sidebands. Then, the first-order comb teeth of the SMC are beaten with the reference sidebands and only the difference frequency is passively filtered out by a PD. The repetition rate of the SMC is locked to the RF reference when the beat frequency approaches to zero. The beat frequency is minimized by carefully feeding back (10 mK per step) the MRR chip temperature with the thermoelectric cooler (TEC). Though the repetition rates of fabricated chips differ from each other with tens of kHz, These differences can be precisely eliminated by precise temperature control. Compared with the microwave injection locking method, this approach achieves active locking, making the SMC more robust while further reducing the reliance on high-speed PD. The TEC-driven repetition rate variations of SMC chips are given in Supplementary Figs. 2b and 2c. These results demonstrate that the repetition rate can be adjusted and stabilized on demand. The active locking scheme eliminates the competitive mechanism and guarantees robust locking of the SMC source, which is a long-term prerequisite for a practical MDI quantum network. The beat frequency of independent RF references is shown in Supplementary Fig. 3. The standard deviation is only 2.7 Hz, much more stable than the fluctuation of repetition rates of SMC chips, and hence can be used as the reference to lock repetition rate. Compared to microdisk cavities (such as MgF_2 cavities) fabricated by polishing (*1, 2*), the SMC chip is fully compatible with CMOS fabrication processes and, hence, the repetition rate of

independent SMC sources can be locked with high precision.

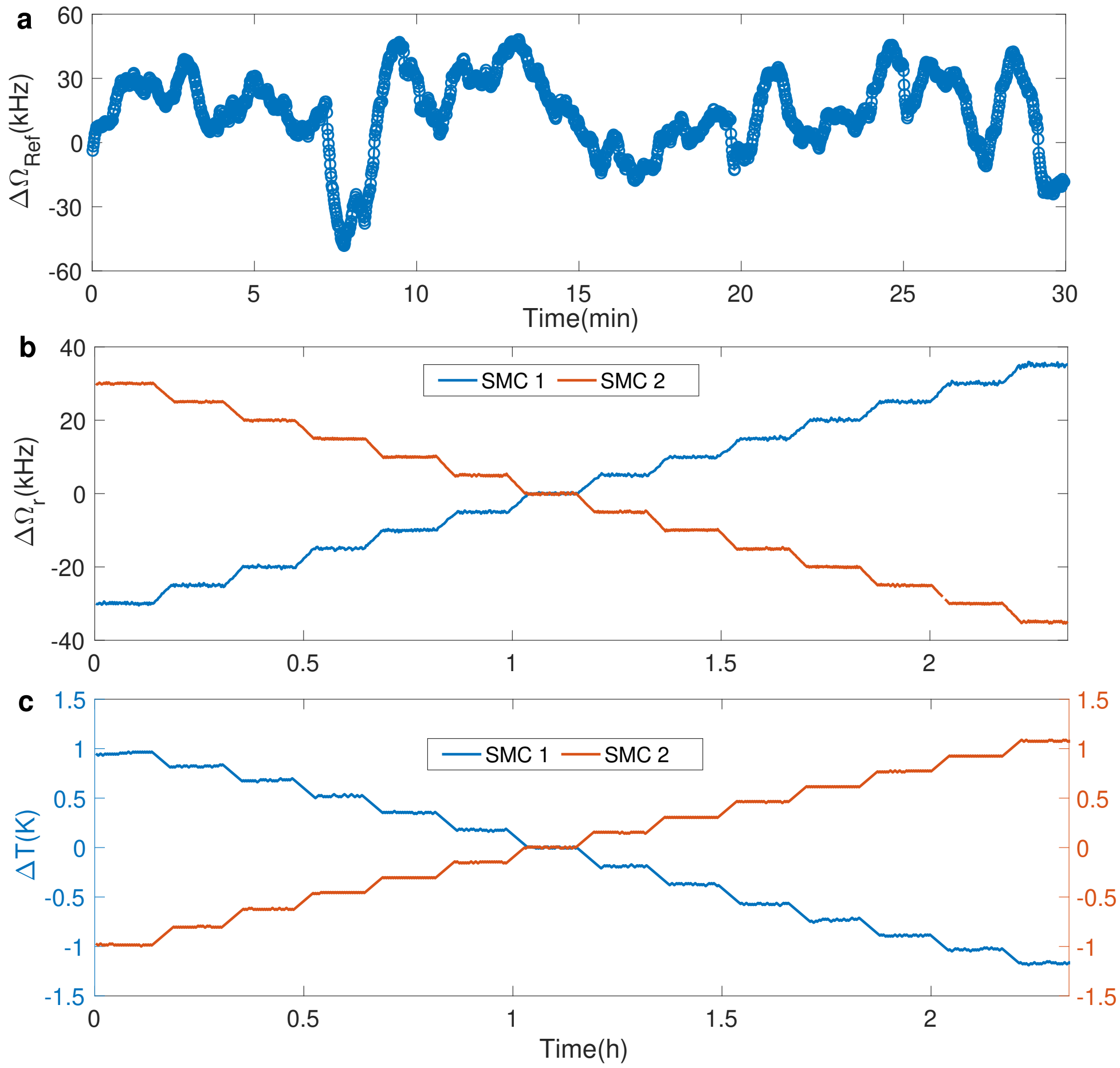

Supplementary Figure 2: **TEC-driven repetition rate variations of the SMC chips. a**, The repetition rate drifts without active locking; **b**, The repetition rate variation and **c**, the driven temperature of the SMC chips under active thermal locking.

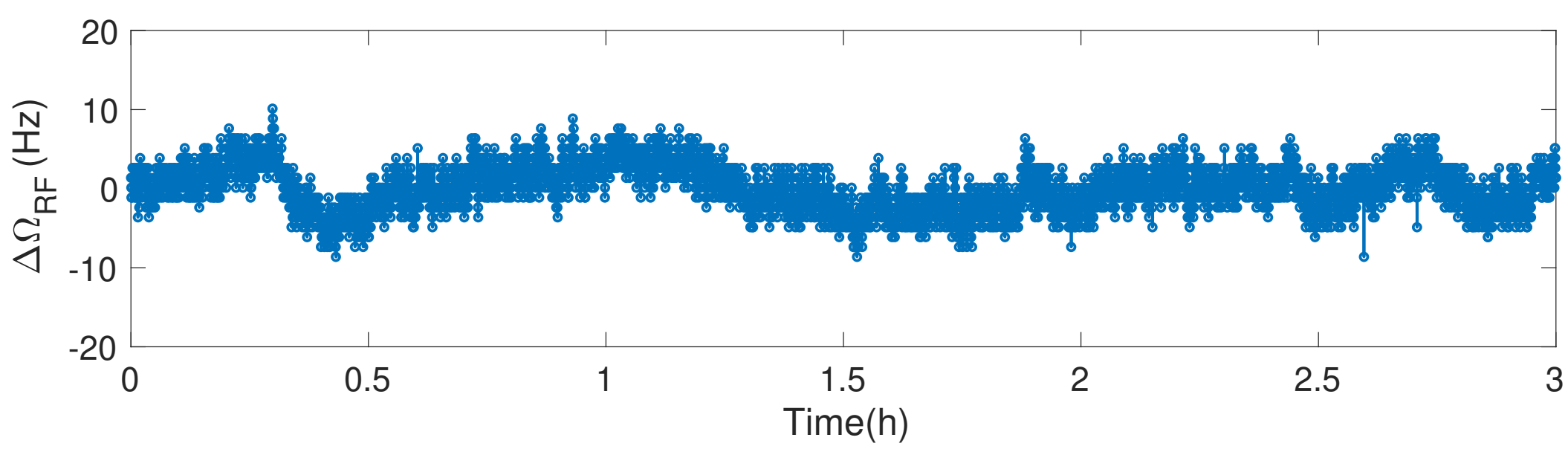

Supplementary Figure 3: **The beat frequency ($\Delta\Omega_{\mathrm{RF}}$) of independent RF sources.**

The photograph of MDI-QKD transmitter chips with optoelectronic package is shown in Sup-

plementary Fig. 1b. A high-performance QKD system requires precise preparation of quantum states. To achieve a high modulation extinction ratio of the MZI, the bias voltage imposed on the carrier-depletion PMs are carefully adjusted to balance the insertion loss of both arms. Each comb tooth is randomly modulated by applying 2.5-GHz analog signals to the PMs. Phase randomization is first performed using a lithium niobate electro-optic phase modulator before being incident into the transmitter chip. All MZIs of the transmitter chips operate in push-pull modulation mode. A 2.5-GHz narrow pulsed signal is applied to MZI-1 to chop the CW comb tooth into narrow pulses. MZI-2 is randomly modulated by a four-amplitude 2.5-GHz signal to prepare decoy states (s, μ, ν, ω). For the Z basis, MZI-3 is randomly modulated by V_0 and V_π, so that the photon pulses are coupled to the upper or down path of MZI-4 only. The upper and lower paths are then mapped into $|H\rangle$ and $|V\rangle$, respectively, across the 2D-GC. For the X basis, the phase difference of MZI-3 is fixed to $\pi/2$, while that of MZI-4 is randomly modulated between $\pi/2$ and $-\pi/2$. Then, the photon pulses are mapped into $|+\rangle$ and $|-\rangle$, respectively.

Supplementary Note 2 – Secure Key Rate Analysis

The MDI-QKD protocol adopts two mutually unbiased bases (MUBs) and a four-intensity decoy-state method (*3,4*). In this method, Alice (Bob) sends the Z basis $\{|H\rangle, |V\rangle\}$ with intensity s_A (s_B), and sends the X-basis $\{|+\rangle, |-\rangle\}$ with intensities μ_A, ν_A, ω_A (μ_B, ν_B, ω_B), where ω_A and ω_B are the vacuum states. The subscript A (B) is used to denote a variable or a parameter that belongs to Alice (Bob). For simplicity, we will omit the subscript A or B if it does not cause any ambiguity. The Z-basis is used to obtain the final keys, while the X basis is used to estimate the yield and phase-flip error of the single-photon pairs in the Z-basis. Due to the phase randomization of the pulses, the density matrices generated by the Alice's and Bob's sources can be expanded in the particle-number space in the following form

$$\begin{aligned} \rho_l &= \sum_j a_j^l |j\rangle\langle j|, l = s_A, \mu_A, \nu_A, \omega_A, \\ \rho_r &= \sum_k b_k^r |k\rangle\langle k|, r = s_B, \mu_B, \nu_B, \omega_B. \end{aligned} \tag{1}$$

Here we assume

$$\frac{a_j^\mu}{a_j^\nu} \geqslant \frac{a_2^\mu}{a_2^\nu} \geqslant \frac{a_1^\mu}{a_1^\nu}, \frac{b_k^\mu}{b_k^\nu} \geqslant \frac{b_2^\mu}{b_2^\nu} \geqslant \frac{b_1^\mu}{b_1^\nu}, \tag{2}$$

where $j > 2$ and $k > 2$. This assumption is the basic requirement for the analytical formula of the decoy state method in this paper. For a weak coherent source, Eq. (2) reduces to

$$\mu_A \geq \nu_A, \mu_B \geq \nu_B. \tag{3}$$

We define N_{lr} as the number of sent pulses using different intensities,

$$N_{lr} = N p_l p_r, \tag{4}$$

where N is the total number of pulses send by Alice and Bob, p_l (p_r) is the probability that Alice (Bob) sends with intensity $l \in \{s_A, \mu_A, \nu_A, \omega_A\}$ ($r \in \{s_B, \mu_B, \nu_B, \omega_B\}$). n_{lr} and m_{lr} are employed to denote the effective gain number and the wrong effective gain number, which are obtained from the experiments. Furthermore, considering the influence of the finite-size effect, we use the Chernoff bound (*5, 6*) to estimate the upper and lower bounds of the fluctuation of the parameters. The Chernoff bound can be employed to estimate the expected values from their observed values or estimating the observed values from their expected values. $\langle n_{lr}\rangle^L$ and $\langle n_{lr}\rangle^U$ are employed to denote the lower and upper bounds of the expected value of $\langle n_{lr}\rangle$. In addition, we can obtain the lower and upper bounds of the expected value of the wrong effective gain number $\langle m_{lr}\rangle$, which are denoted as $\langle m_{lr}\rangle^L$ and $\langle m_{lr}\rangle^U$. The bounds of the expected values are estimated from the measured values n_{lr} and m_{lr}.

For a higher key rate, the double-scanning method (*7*) is used. The lower bound of the yield and the upper bound of the phase-flip error rate of the single-photon pairs in the Z-basis, $y^L_{11,Z}$ and $e^{p,U}_{11,Z}$, are crucial parameters in the process of calculating the final key rate. As shown in Ref. (*3*),

$$\begin{aligned} \langle y_{11,X}\rangle =& \langle y_{11,Z}\rangle, \\ \langle e^b_{11,X}\rangle =& \langle e^p_{11,Z}\rangle, \end{aligned} \tag{5}$$

where $\langle y_{11,X}\rangle$ and $\langle y_{11,Z}\rangle$ are the expected values of the yield in the X- and Z-basis, and $\langle e^b_{11,X}\rangle$ ($\langle e^p_{11,Z}\rangle$) is the bit-flip (phase-flip) error rate of the single-photon pairs in the X (Z) basis. We can estimate $y^L_{11,Z}$ and $e^{p,U}_{11,Z}$ using $\langle y_{11,X}\rangle^L$ and $\langle e^b_{11,X}\rangle^U$ with the Chernoff bound,

$$y^L_{11,Z} = \frac{O^L(N_{ss} a_1^\mu b_1^\mu \langle y_{11,X}\rangle^L, \xi^L_{y_{11}})}{N_{\mu\mu} a_1^\mu b_1^\mu}, \tag{6}$$

$$e^{p,U}_{11,Z} = \frac{O^U(N_{ss} a_1^\mu b_1^\mu y^L_{11,Z} \langle e^b_{11,X}\rangle^U, \xi^U_{e_{11}})}{N_{\mu\mu} a_1^\mu b_1^\mu y^L_{11,Z}}, \tag{7}$$

where $O^L(E,\xi)$ and $O^U(E,\xi)$ are the lower and upper bounds of the observed value O, whose expected value is E. Our purpose is to calculate $\langle y_{11,X}\rangle^L$ and $\langle e^b_{11,X}\rangle^U$. According to the formulas in Ref. (*7*), if $\frac{\mu_B}{\nu_B} \leq \frac{\mu_A}{\nu_A}$, we have

$$\left\langle y_{11,X}\right\rangle^L = \frac{\langle Q_+\rangle^L + \frac{a_1^\mu b_2^\mu}{N_{\nu\nu}}\mathcal{M} - \langle Q_-\rangle^U - a_1^\mu b_2^\mu \mathcal{H}}{a_1^\nu a_1^\mu \left(b_1^\nu b_2^\mu - b_2^\nu b_1^\mu\right)}, \tag{8}$$

$$\left\langle e^b_{11,X}\right\rangle^U = \frac{\frac{1}{N_{\nu\nu}}\mathcal{M} - \frac{1}{2}\mathcal{H}}{a_1^\nu b_1^\nu \left\langle y_{11,X}\right\rangle^L}, \tag{9}$$

where

$$\langle Q_+\rangle = \frac{a_1^\mu b_2^\mu}{N_{\nu\nu}}\langle \bar{m}_{\nu\nu}\rangle + \frac{a_1^\nu b_2^\nu a_0^\mu}{N_{\omega\mu}}\left\langle n_{\omega\mu}\right\rangle + \frac{a_1^\nu b_2^\nu b_0^\mu}{N_{\mu\omega}}\left\langle n_{\mu\omega}\right\rangle, \tag{10}$$

$$\langle Q_-\rangle = \frac{a_1^\nu b_2^\nu}{N_{\mu\mu}}\left\langle n_{\mu\mu}\right\rangle + \frac{a_1^\nu b_2^\nu a_0^\mu b_0^\mu}{N_{\omega\omega}}\langle n_{\omega\omega}\rangle, \tag{11}$$

$$\mathcal{H} = \frac{a_0^\nu}{N_{\omega\nu}}\langle n_{\omega\nu}\rangle + \frac{b_0^\nu}{N_{\nu 0}}\langle n_{\nu\omega}\rangle - \frac{a_0^\nu b_0^\nu}{N_{\omega\omega}}\langle n_{\omega\omega}\rangle, \tag{12}$$

$$\mathcal{M} = \langle m_{\nu\nu}\rangle. \tag{13}$$

$\langle \bar{m}_{\nu\nu}\rangle$ in Eq.(10) is the expected value of the number of right events of the intensity $\nu\nu$, which is

$$\langle \bar{m}_{\nu\nu}\rangle = \langle n_{\nu\nu}\rangle - \langle m_{\nu\nu}\rangle. \tag{14}$$

In addition, in the case of $\frac{\mu_B}{\nu_B} > \frac{\mu_A}{\nu_A}$, $\langle y_{11,X}\rangle^L$ can be obtained with Eq. (8) by making the exchange between a_k^μ and b_k^μ, and the exchange between a_k^ν and b_k^ν, for $k = 1, 2$. $\langle e^b_{11,X}\rangle^U$ is obtained similarly.

From the experiments we obtain the observed values, but Eq. (8) and Eq. (9) involve only expected values. $E^L(O,\xi)$ and $E^U(O,\xi)$ are the lower and upper bounds of the expected value E, which are estimated from the observed value O. To obtain tight estimates of $\left\langle y_{11,X}\right\rangle^L$ and $\left\langle e^b_{11,X}\right\rangle^U$, we use the joint constraints methods in Ref. (*8*). In this method, we can get

$$\langle Q_+\rangle^L = F_L\Big(\frac{a_1^\mu b_2^\mu}{N_{\nu\nu}}, \frac{a_1^\nu b_2^\nu a_0^\mu}{N_{\omega\mu}}, \frac{a_1^\nu b_2^\nu b_0^\mu}{N_{\mu\omega}}, \bar{m}_{\nu\nu}, n_{\omega\mu}, n_{\mu\omega}, \xi^L_{Q_1^+}, \xi^L_{Q_2^+}, \xi^L_{Q_3^+}\Big), \tag{15}$$

$$\langle Q_-\rangle^U = F_U\Big(\frac{a_1^\nu b_2^\nu}{N_{\mu\mu}}, \frac{a_1^\nu b_2^\nu a_0^\mu b_0^\mu}{N_{\omega\omega}}, 0, n_{\mu\mu}, n_{\omega\omega}, 0, \xi^U_{Q_1^-}, \xi^U_{Q_2^-}, 0\Big), \tag{16}$$

$$\mathcal{H}^L = F_L\Big(\frac{a_0^\nu}{N_{\omega\nu}}, \frac{b_0^\nu}{N_{\nu\omega}}, 0, n_{\omega\nu}, n_{\nu\omega}, 0, \xi_{H_1}^L, \xi_{H_2}^L, 0\Big) - \frac{a_0^\nu b_0^\nu}{N_{\omega\omega}} E^U(n_{\omega\omega}, \xi_{H_3}^L),$$
$$\mathcal{H}^U = F_U\Big(\frac{a_0^\nu}{N_{\omega\nu}}, \frac{b_0^\nu}{N_{\nu\omega}}, 0, n_{\omega\nu}, n_{\nu\omega}, 0, \xi_{H_1}^U, \xi_{H_2}^U, 0\Big) - \frac{a_0^\nu b_0^\nu}{N_{\omega\omega}} E^L(n_{\omega\omega}, \xi_{H_3}^U), \quad (17)$$

$$\mathcal{M}^L = E^L(m_{\nu\nu}, \xi_M^L), \mathcal{M}^L = E^U(m_{\nu\nu}, \xi_M^U). \quad (18)$$

With the equations above all, we have

$$R = p_{s_A} p_{s_B} \{a_1^s b_1^s y_{11,Z}^L [1 - h(e_{11}^{p,U})] - f_e Q_{ss} h(E_{ss})\}$$
$$- \frac{1}{N}\left(\log_2 \frac{8}{\varepsilon_{cor}} + 2\log_2 \frac{2}{\varepsilon' \hat{\varepsilon}} + 2\log_2 \frac{1}{2\varepsilon_{PA}}\right), \quad (19)$$

where $Q_{ss} = n_{ss}/N_{ss}$ is the gain of the pulse pairs in the Z-basis; E_{ss} is the QBER in the Z-basis; $h(x) = -x\log_2 x - (1-x)\log_2(1-x)$ is the Shannon entropy; f_e is the efficiency factor of the error correction method; ε_{cor} is the failure probability of error correction; ε_{PA} is the failure probability of privacy amplification; ε' and $\hat{\varepsilon}$ are the coefficients used in the chain rules of smooth min and max entropy.

Eq. (8) and Eq. (9) both contain $\mathcal{H}$ and $\mathcal{M}$, which means there is a correlation between $\langle y_{11,X}\rangle^L$ and $\langle e_{11,X}^b\rangle^U$. By scanning $\mathcal{H}$ and $\mathcal{M}$,

$$R = \min_{\mathcal{H},\mathcal{M}} R(\mathcal{H},\mathcal{M}), \quad (20)$$

where $\mathcal{H} \in [\mathcal{H}^L, \mathcal{H}^U]$ and $\mathcal{M} \in [\mathcal{M}^L, \mathcal{M}^U]$. The total secure coefficient ε_{tol} is (*9–11*)

$$\varepsilon_{tol} = \varepsilon_{cor} + 2(\varepsilon' + \hat{\varepsilon} + 2\sqrt{\varepsilon_e + \varepsilon_1}) + \varepsilon_{PA}, \quad (21)$$

where $\varepsilon_e = \xi_{e_{11}}^U$ is the probability that the real value of the phase-flip error rate of the effective events of single-photon pairs in the Z-basis is larger than its estimated value $e_{11}^{p,U}$, and ε_1 is the probability that the real value of the counting rate of the single-photon pairs in the Z-basis is less than its estimated value $y_{11,Z}^L$; it is composed of different parts,

$$\varepsilon_1 = \xi_{Q_1^+}^L + \xi_{Q_2^+}^L + \xi_{Q_3^+}^L + \xi_{Q_1^-}^U + \xi_{Q_2^-}^U + \xi_{H_1}^L + \xi_{H_2}^L + \xi_{H_3}^L + \xi_{H_1}^U + \xi_{H_2}^U + \xi_{H_3}^U + \xi_M^L + \xi_M^U + \xi_{y_{11}}. \quad (22)$$

Taking Eq. (10) as an example, by the method of joint constraints , $\langle S_+\rangle^L$ can be obtained with constraints:

$$
\begin{aligned}
&\langle \bar{m}_{\nu\nu}\rangle \geq E^L(\bar{m}_{\nu\nu}, \xi),\\
&\langle n_{\omega\mu}\rangle \geq E^L(n_{\omega\mu}, \xi),\\
&\langle n_{\mu\omega}\rangle \geq E^L(n_{\mu\omega}, \xi),\\
&\langle \bar{m}_{\nu\nu}\rangle + \langle n_{\omega\mu}\rangle \geq E^L(\bar{m}_{\nu\nu} + n_{\omega\mu}, \xi),\\
&\langle n_{\omega\mu}\rangle + \langle n_{\mu\omega}\rangle \geq E^L(n_{\omega\mu} + n_{\mu\omega}, \xi),\\
&\langle \bar{m}_{\nu\nu}\rangle + \langle n_{\mu\omega}\rangle \geq E^L(\bar{m}_{\nu\nu} + n_{\mu\omega}, \xi),\\
&\langle \bar{m}_{\nu\nu}\rangle + \langle n_{\omega\mu}\rangle + \langle n_{\mu\omega}\rangle \geq E^L(\bar{m}_{\nu\nu} + n_{\omega\mu} + n_{\mu\omega}, \xi).
\end{aligned} \tag{23}
$$

This problem can be solved by linear programming, but this approach would be time-consuming, especially when we optimize the parameters to achieve the highest key rate. Therefor, an analytical result of this special linear programming problem is necessary. It should be noted that the failure probability in estimating $\langle S_+\rangle^L$ with this method is 3ξ, because at most three of the constraints are used in the final results. To obtain an analytic result, the problem can be abstracted into the form given in (*7, 8*)

$$
\begin{aligned}
&\min_{g_1,g_2,g_3} F = \gamma_1 g_1 + \gamma_2 g_2 + \gamma_3 g_3,\\
&s.t. \quad g_1 \geq E^L(\widetilde{g_1}, \xi_1),\\
&\qquad g_2 \geq E^L(\widetilde{g_2}, \xi_1),\\
&\qquad g_3 \geq E^L(\widetilde{g_3}, \xi_1),\\
&\qquad g_1 + g_2 \geq E^L(\widetilde{g_1} + \widetilde{g_2}, \xi_2),\\
&\qquad g_2 + g_3 \geq E^L(\widetilde{g_2} + \widetilde{g_3}, \xi_2),\\
&\qquad g_1 + g_3 \geq E^L(\widetilde{g_1} + \widetilde{g_3}, \xi_2),\\
&\qquad g_1 + g_2 + g_3 \geq E^L(\widetilde{g_1} + \widetilde{g_2} + \widetilde{g_3}, \xi_3),
\end{aligned} \tag{24}
$$

where $\gamma_1, \gamma_2, \gamma_3, g_1, g_2, g_3, \widetilde{g_1}, \widetilde{g_2}, \widetilde{g_3}$ are all positive.

We rearrange $\{\gamma_1, \gamma_2, \gamma_3\}$ in ascending order; the new sequence is denoted by $\{\gamma_1', \gamma_2', \gamma_3'\}$ and $\{\widetilde{g_1}', \widetilde{g_2}', \widetilde{g_3}'\}$ denotes the corresponding rearrangement of $\{\widetilde{g_1}, \widetilde{g_2}, \widetilde{g_3}\}$ according to the ascending

order of $\{\gamma_1, \gamma_2, \gamma_3\}$. In this way, the lower bound of F can be written as:

$$\begin{aligned} &F_L(\gamma_1, \gamma_2, \gamma_3, \widetilde{g_1}, \widetilde{g_2}, \widetilde{g_3}, \xi_1, \xi_2, \xi_3) \\ =&\gamma_1' E^L(\widetilde{g_1}' + \widetilde{g_2}' + \widetilde{g_3}', \xi_3) + (\gamma_2' - \gamma_1')E^L(\widetilde{g_2}' + \widetilde{g_3}', \xi_2) + (\gamma_3' - \gamma_2')E^L(\widetilde{g_3}', \xi_1). \end{aligned} \tag{25}$$

To obtain the upper bound of F, we can simply replace $E^L(O, \xi)$ with $E^U(O, \xi)$:

$$\begin{aligned} &F_U(\gamma_1, \gamma_2, \gamma_3, \widetilde{g_1}, \widetilde{g_2}, \widetilde{g_3}, \xi_1, \xi_2, \xi_3) \\ =&\gamma_1' E^U(\widetilde{g_1}' + \widetilde{g_2}' + \widetilde{g_3}', \xi_3) + (\gamma_2' - \gamma_1')E^U(\widetilde{g_2}' + \widetilde{g_3}', \xi_2) + (\gamma_3' - \gamma_2')E^U(\widetilde{g_3}', \xi_1). \end{aligned} \tag{26}$$

The Chernoff bound (5) is useful for estimating expected values from observed values, or estimating observed values from expected values. Let $X_1, X_2, ..., X_n$ be a set of independent Bernoulli random variables (which may follow different distributions), and let $X = \sum_{i=1}^{n} X_i$. The observed value of X, denoted by O, is unknown and its expected value E is known. In this situation, we have

$$O^L(E, \xi) = [1 - \delta_1(E, \xi)]E, \tag{27}$$

$$O^U(E, \xi) = [1 + \delta_2(E, \xi)]E, \tag{28}$$

where $\delta_1(E, \xi)$ and $\delta_2(E, \xi)$ can be obtained by solving the following equations:

$$\left(\frac{e^{-\delta_1}}{(1-\delta_1)^{1-\delta_1}}\right)^E = \xi, \tag{29}$$

$$\left(\frac{e^{\delta_2}}{(1+\delta_2)^{1+\delta_2}}\right)^E = \xi, \tag{30}$$

where ξ is the failure probability.

When the expected value E is unknown and its observed value O is known, we have

$$E^L(O, \xi) = \frac{O}{1 + \delta_1'(O, \xi)}, \tag{31}$$

$$E^U(O, \xi) = \frac{O}{1 - \delta_2'(O, \xi)}, \tag{32}$$

where $\delta_1'(O, \xi)$ and $\delta_2'(O, \xi)$ can be obtained by solving the following equations:

$$\left(\frac{e^{\delta_1'}}{(1+\delta_1')^{1+\delta_1'}}\right)^{\frac{O}{1+\delta_1'}} = \xi, \tag{33}$$

$$\left(\frac{e^{-\delta_2'}}{(1-\delta_2')^{1-\delta_2'}}\right)^{\frac{O}{1-\delta_2'}} = \xi. \tag{34}$$

Supplementary Note 3 – Polarization Stability and Feedback for Long-Distance fibre

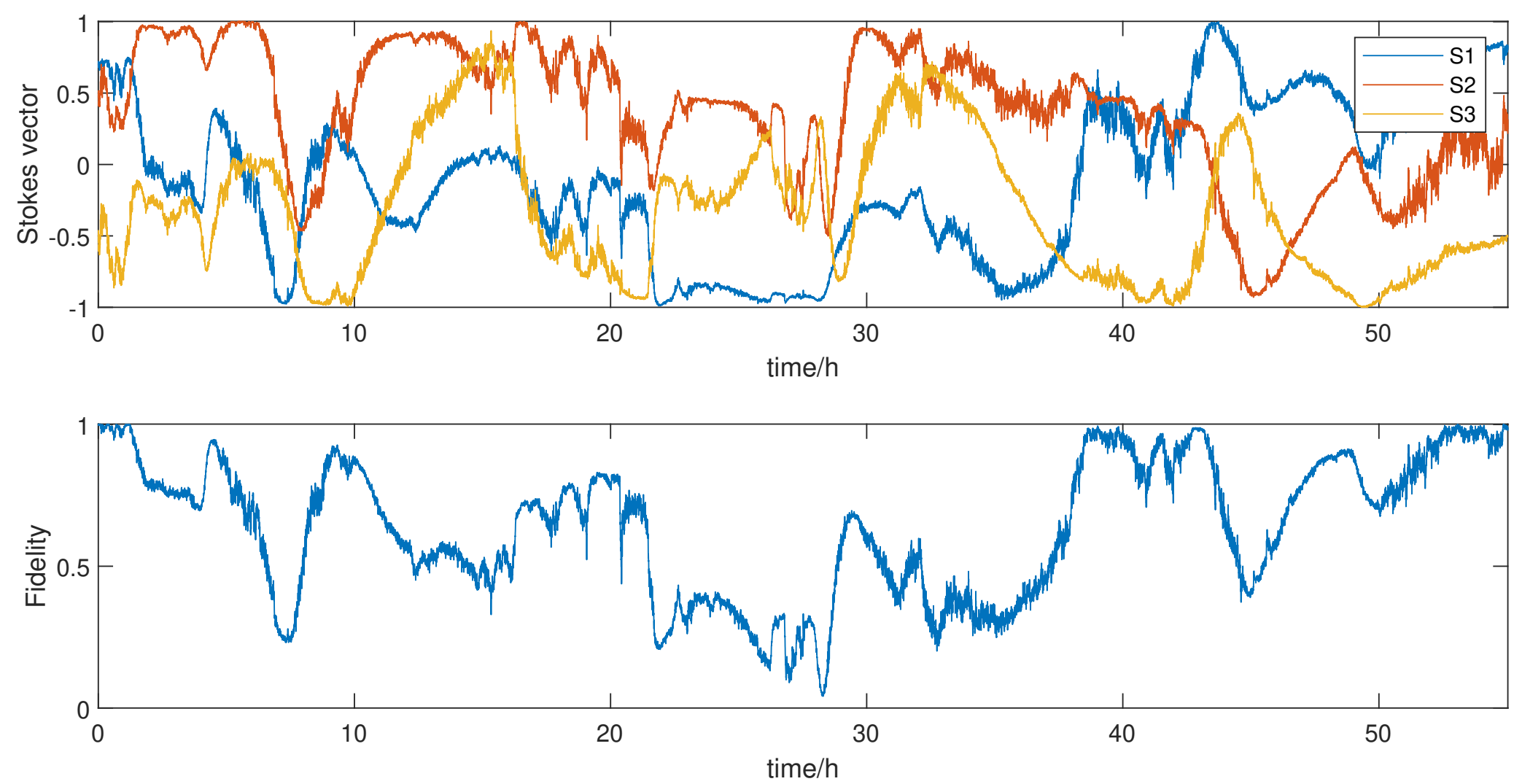

Supplementary Figure 4: **Stokes vector and polarization fidelity. Top**, Stokes vector of emitted light from a 100-km fibre spool in the laboratory over 55 hours. **Bottom**, Polarization fidelity with respect to the initial time.

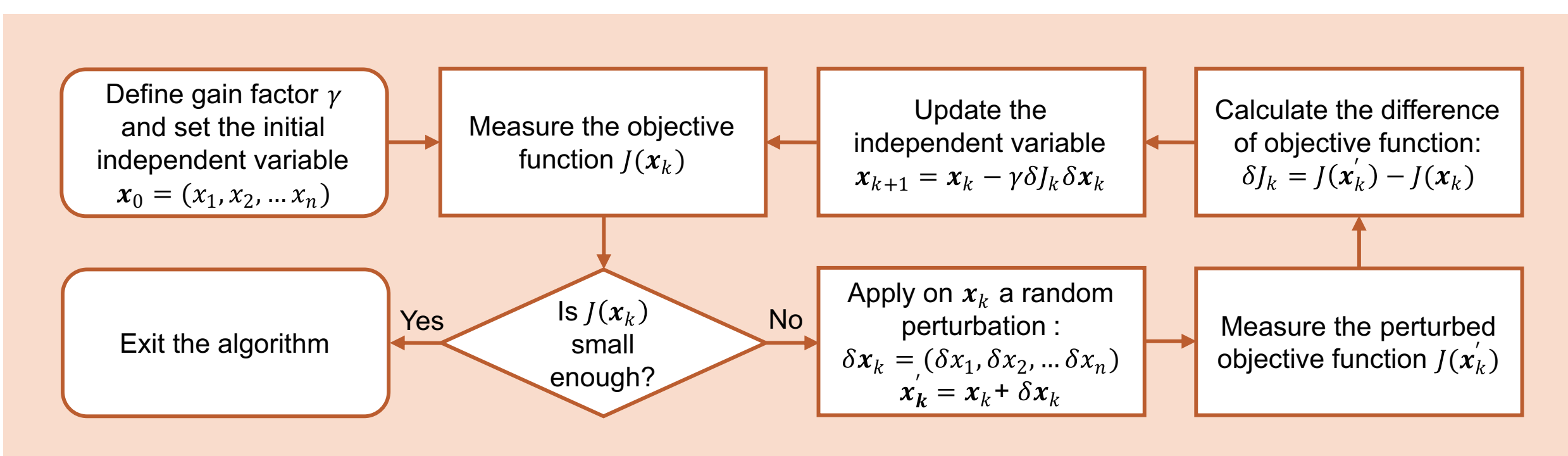

Supplementary Figure 5: **Flowchart of the SPGD algorithm.**

Polarization dynamically varies through long-distance fibre and hence degrades the photon fidelity and the HOM interference visibility. Supplementary Fig. 4 shows the polarization drift of a 100-km fibre spool in the lab environment, where the fidelity of the input polarization degrades over time and hinders the long-time secure key distribution of polarization-encoded QKD systems.

Thus, dynamic polarization compensation is vital for QKD. The stochastic parallel gradient descent (SPGD) (*12*) algorithm is an effective tool to compensate polarization variations in fibre. As exhibited in Supplementary Fig. 5, to minimize the objective function $J(\boldsymbol{x})$ with independent variable $\boldsymbol{x_k} = (x_1, x_2, ..., x_n)$, a random perturbation $\delta\boldsymbol{x}_k$ is applied on $\boldsymbol{x}_k$ such that $\boldsymbol{x}'_k = \boldsymbol{x}_k + \delta\boldsymbol{x}_k$, in each round. Then the perturbed objective function $J(\boldsymbol{x}'_k)$ measured and its perturbation is calculated as $\delta J_k = J(\boldsymbol{x}'_k) - J(\boldsymbol{x}_k)$. Finally, the independent variable is updated as $\boldsymbol{x}_{k+1} = \boldsymbol{x}_k - \gamma\delta J_k \delta\boldsymbol{x}_k$ (γ is the gain factor). The polarization is iteratively optimized until the objective function is lower than a the given threshold. SPGD-based polarization compensation ensures low QBERs in, for example, polarization-encoded BB84 (*13*) or time-phase-encoded QKDs (*14*). Unlike the BB84 protocol, the X basis of polarization-encoded MDI-QKD can only be calibrated via two-photon coincidence of anti-diagonal polarization states. The low coincidence counts of the X basis give rise to significant statistical fluctuations. On the other hand, the alignment of both Z and X bases between Alice, Bob and Charlie must be maintained simultaneously. This corresponds to an isolated point solution and brings seemingly insurmountable technical challenges. Thus, long-fibre drift compensation for polarization-based MDI-QKD has not been achieved so far (*15*).

We propose a two-stage feedback scheme to overcome the low coincidence counts of the X basis. A small portion of the photon sequence are used as calibration states. For the Z basis, the calibration states are arranged as $\{s^{\text{Alice}}, \omega^{\text{Bob}}\}$ ($\{\omega^{\text{Alice}}, s^{\text{Bob}}\}$) and the single-detector clicks are used to compensate the polarization drifts between Alice (Bob) and Charlie. To estimate the misalignment of the X basis, the calibration states consist of all possible combinations of the X basis and the average QBER of coincidence counts is used to compensate the misalignment of polarization. The compensation is realized by electrically driven polarization controllers (EPCs). The compensation of the Z and X bases is carried out alternately. As the coincidence count rate is much smaller than the single-detector click rate, the calibration accumulation period of the X basis is longer than that of the Z basis.

The compensation periods for the Z and X bases are 5 s and 40 s, respectively. The calibration states are randomly merged into the QKD encoding states and the intensity is the same as that of the signal state s. The sending probabilities of calibration states are substantially suppressed to guarantee the secure key distribution efficiency. The Z basis calibration states $\{s^{\text{Alice}}, \omega^{\text{Bob}}\}$ and $\{\omega^{\text{Alice}}, s^{\text{Bob}}\}$ only consume 0.5% of the system's operation time, respectively. The calibration pro-

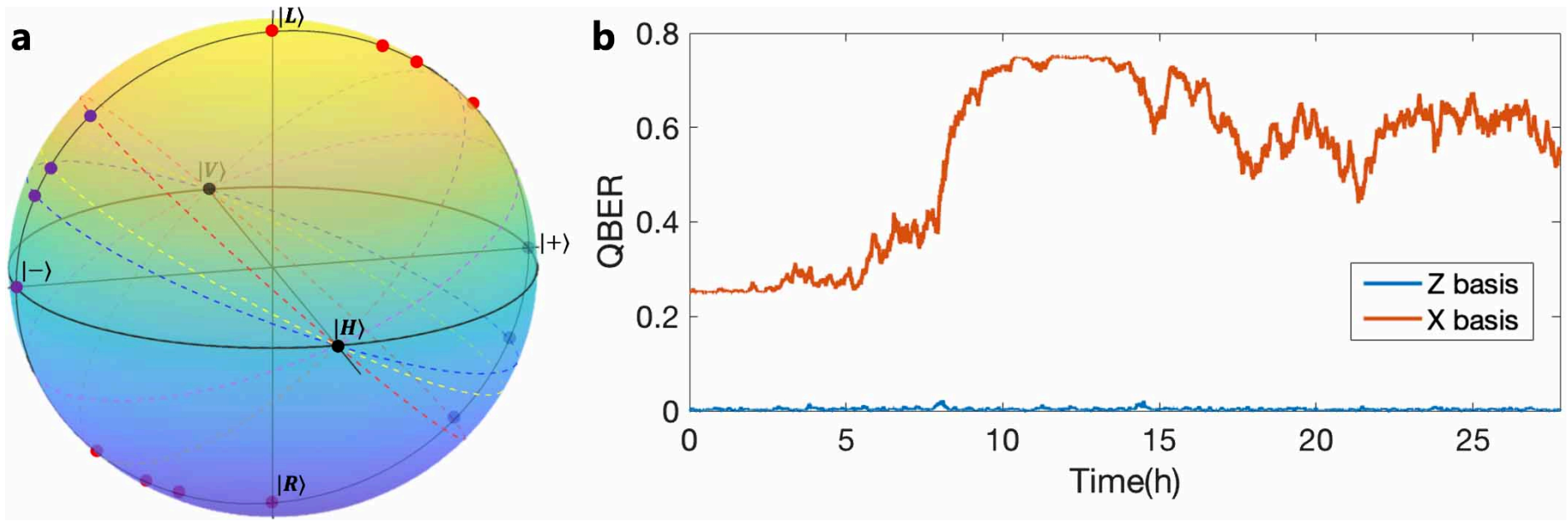

Supplementary Figure 6: **One-stage polarization compensation. a**, Uncontrolled polarization rotation of X and Y bases and **b**, A typical simulated QBER evolution for one-stage polarization compensation with the Z basis fixed.

cess of the X basis consumes 2% of the system's operation time. Considering the finite accumulation time of calibration states, the statistical QBER fluctuations of the Z and X bases are about 0.036% and 0.58%, respectively, for the 200-km MDI-QKD system. Thus, the fluctuation errors due to finite accumulation time are negligible.

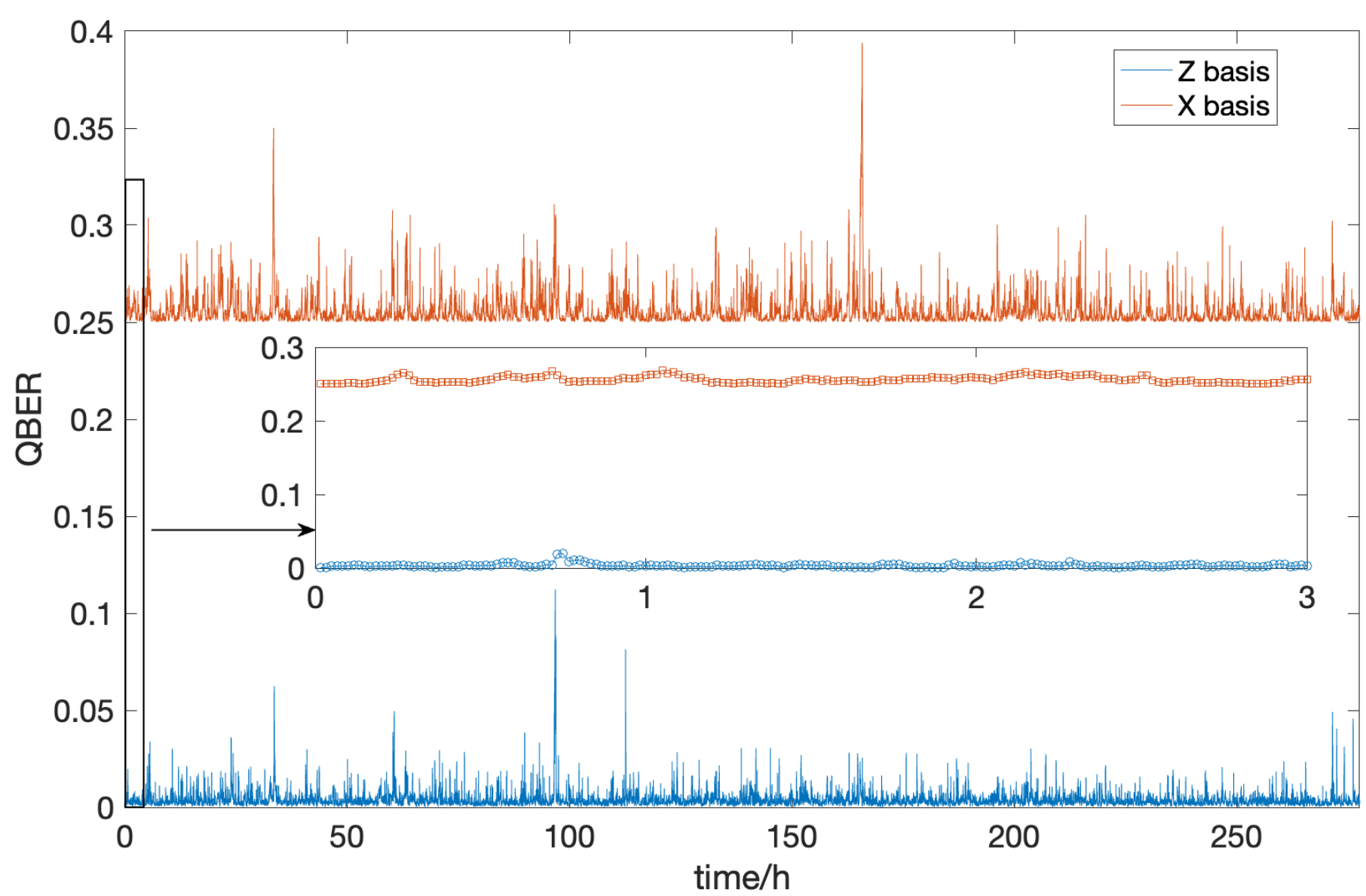

Supplementary Figure 7: **Two-stage polarization compensation.** A typical simulated QBER evolution with two-stage polarization compensation.

To verify the long-time stability of the SPGD-based polarization compensation, we simulate the feedback process with two independent 100-km fibres for ~280 hours. The polarization drift

speed (*16*) and intrinsic QBERs are similar to the experimental MDI-QKD system, respectively. It should be noted that the alternate compensation strategy suppresses the random drifts of polarization, because any drift of the X (Z) basis around the compensation axis of the Z (X) basis is compensated automatically. As shown in Supplementary Fig. 6a, when only the Z basis is compensated, the X and Y bases are totally uncontrolled and rotate around the axis of the Z basis. Hence, the QBER of the X basis is relatively stable initially and becomes out of control as the polarization drift accumulates (Supplementary Fig. 6b).

The two-stage compensation results are exhibited in Supplementary Fig. 7. By adjusting the feedback parameters carefully, we keep increase due to polarization drifts within about 0.4% (Z basis) and 0.6% (X basis), respectively. These results demonstrate a precise polarization alignment method based on a two-stage feedback strategy. Another strategy is to execute only the one-stage feedback for the Z basis in real time, while stopping the QKD system and calibrating the X basis when its QBER exceeds a preset threshold. In fact, the polarization drifting speed of deployed fibre is relatively slow in most situations. In our experiment, setting the QBER threshold of the X basis to 29.5%, the MDI-QKD system can operate continuously for hours.

Supplementary Note 4 – Clock Synchronization and Time Drift Compensation

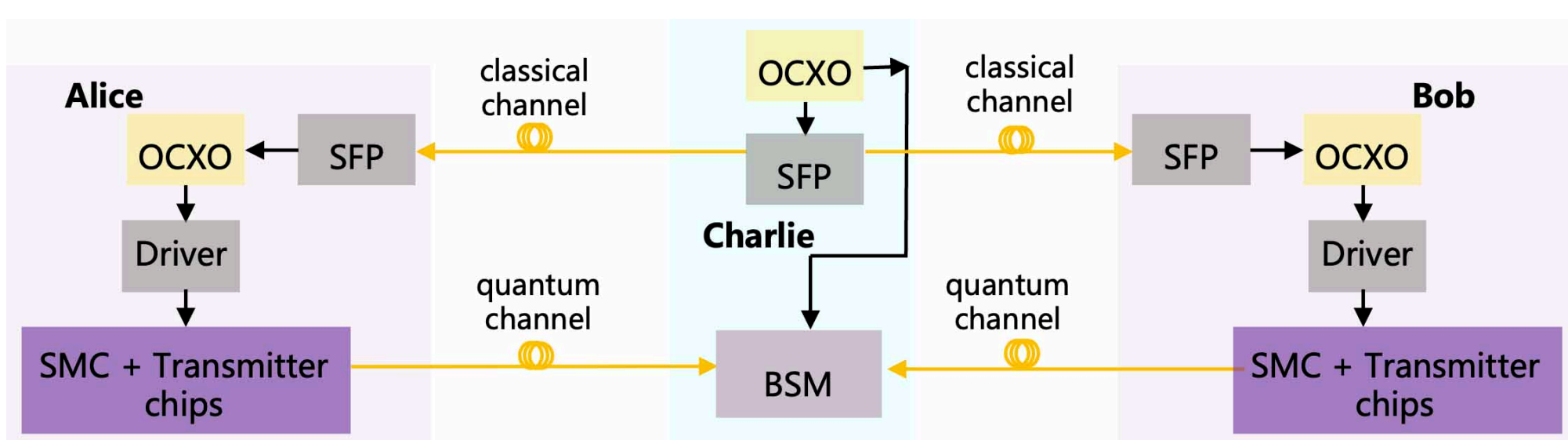

Supplementary Figure 8: **Conceptual setup of the clock synchronization.** SFP, Small form-factor Pluggable Plus; OCXO, oven-controlled crystal oscillator.

Clock synchronization is a prerequisite for establishing a large-scale quantum communication network. The clock synchronization setup is illustrated in Supplementary Fig. 8. Charlie sends his clock to all end users via separate fibres. All clocks are derived from their own oven-controlled crystal oscillators (OCXOs) and synchronized to the clock of Charlie. In fact, the clocks can be

derived from the Rubidium cell and the frequency comb teeth, which is more stable than the OCXO. In such a proposal, additional clocks would be unnecessary. Under this vision, all users could even synchronize their clocks via single-photon-level signals, for example, through two-photon HOM interferences at Charlie. Given the time-frequency synchronization is not the core goal of our work, we have opted for the conventional clock synchronization scheme.

The high visibility of two-photon interference is a prerequisite for guaranteeing a low QBER in the X basis, which means the arrival times of the two narrow pulses from Alice and Bob at Charlie should be precisely aligned. However, environmental changes cause variations in the length of the long-distance fibre, resulting in temporal misalignment between these pulses. The arrival times t_i^A (the count events when Bob sends vacuum states) of pulsed photons from Alice to Charlie are detected and recorded by a multi-channel superconducting nanowire single-photon detector (SNSPD) and a commercial time-to-digital converter (TDC) with a resolution of 1 ps. Only the remainder of t_i^A divided by 400 ps is preserved. The average value of (t_i^A mod 400) is calculated every second, namely, $< t_i^A >= (t_i^A \bmod 400)$. The same operation is performed for the pulsed photons from Bob to Charlie, yielding t_i^B. As the effective count rate per second from Alice (Bob) to Charlie exceeds 2 Mbps, the average arrival time can be precisely estimated. Alice then adjusts the pulse delay accordingly to compensate for the temporal drift of the long-distance fibre. We keep the arrival time difference between Alice and Bob below 2 ps, which is sufficient to guarantee the high visibility of HOM interference.

Supplementary Note 5 – Crosstalk in Frequency Division Multiplexing Channels

The channel crosstalk of the massively multiplexed SMC source is evaluated. Supplementary Fig. 9a shows the evaluation setup. The comb tooth is filtered out (the FWHM of the passband is 0.2 nm) and analyzed by a spectrum analyzer. The crosstalk levels of different channels are mostly less than -30 dB (Supplementary Figs. 9b-9c). The slightly higher of crosstalk of channel H50 is due to the relatively low power of that comb tooth. This can be easily resolved by amplifying the low-power comb teeth.

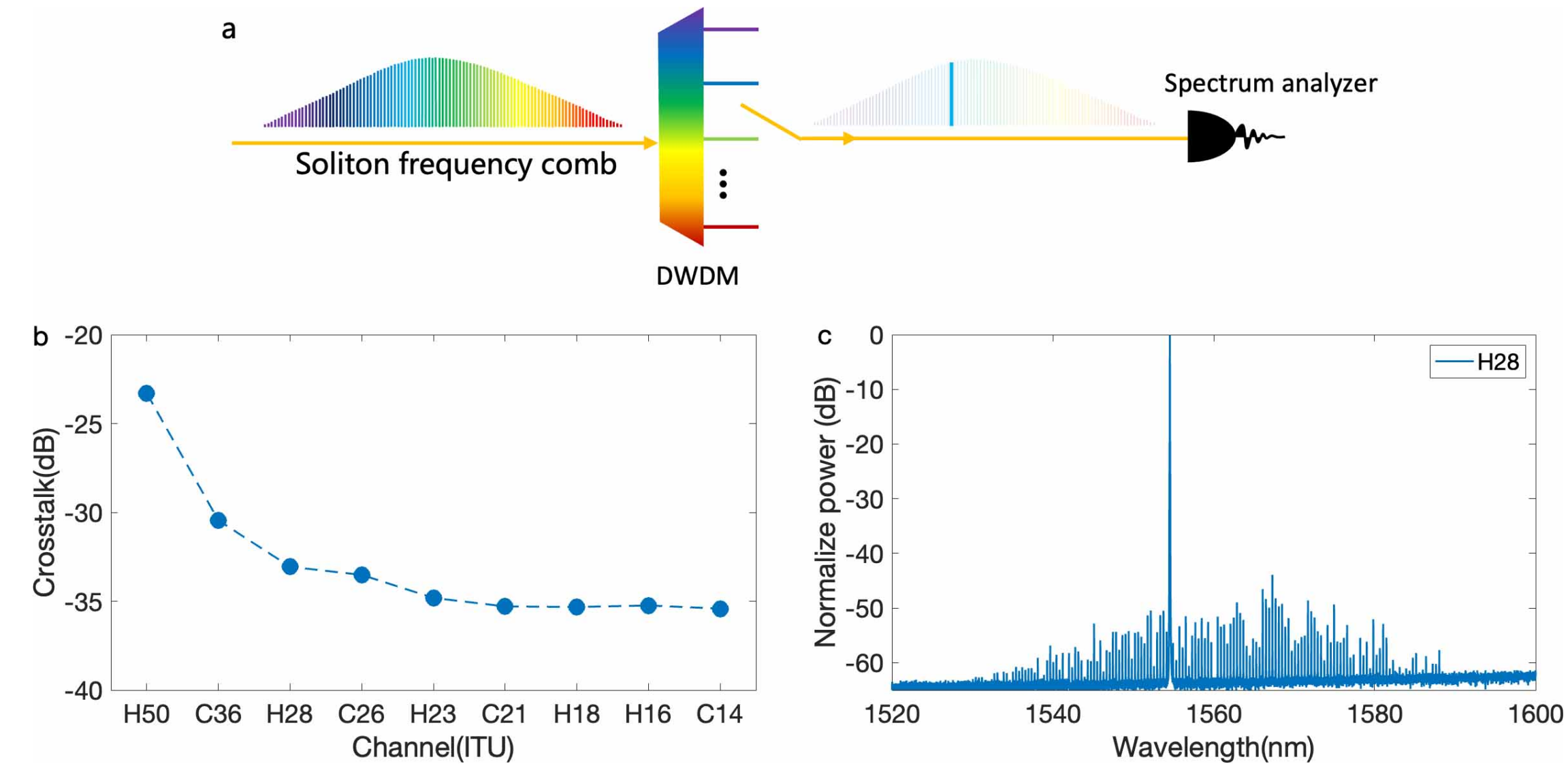

Supplementary Figure 9: **HOM interference of different frequency comb-tooth pairs. a**, Setup, **b**, Crosstalk between different channels and **c**, The power intensity distribution of channel H28 (CH-15) after frequency filtering.

Supplementary Note 6 – HOM Interference Results of Different Channels

The HOM interference visibilities and dips of different SMC tooth pairs are shown in Supplementary Fig. 10. All comb teeth are transmitted through the same fibre channel so that potential crosstalk from a large-scale fully connected MDI quantum network can be emulated (Supplementary Fig. 10a). As demonstrated in Supplementary Fig. 9, the crosstalk between different frequency channels is slight and does not deteriorate the HOM interference visibilities. By compensating for the time delay between narrow pulses (with a FWHM of 95 ps) generated by Alice and Bob's chips and the polarization drifts over distant fibre channels, all channels exhibit high-quality of HOM interference (Supplementary Fig. 10b). The HOM interference dips are measured with a time delay precision of 10 ps and the dips of different tooth pairs are given in Supplementary Figs. 10c-10m.

Supplementary Note 7 – Experimental Data

The detailed experimental results of the microcomb-based MDI-QKD over multiple channels across 200 km are given in Supplementary Table 1. The detailed experimental results of long-time SMC-based MDI-QKD of channel H28 (CH-15 of the SMC source) over a 200-km fibre and a 36-dB

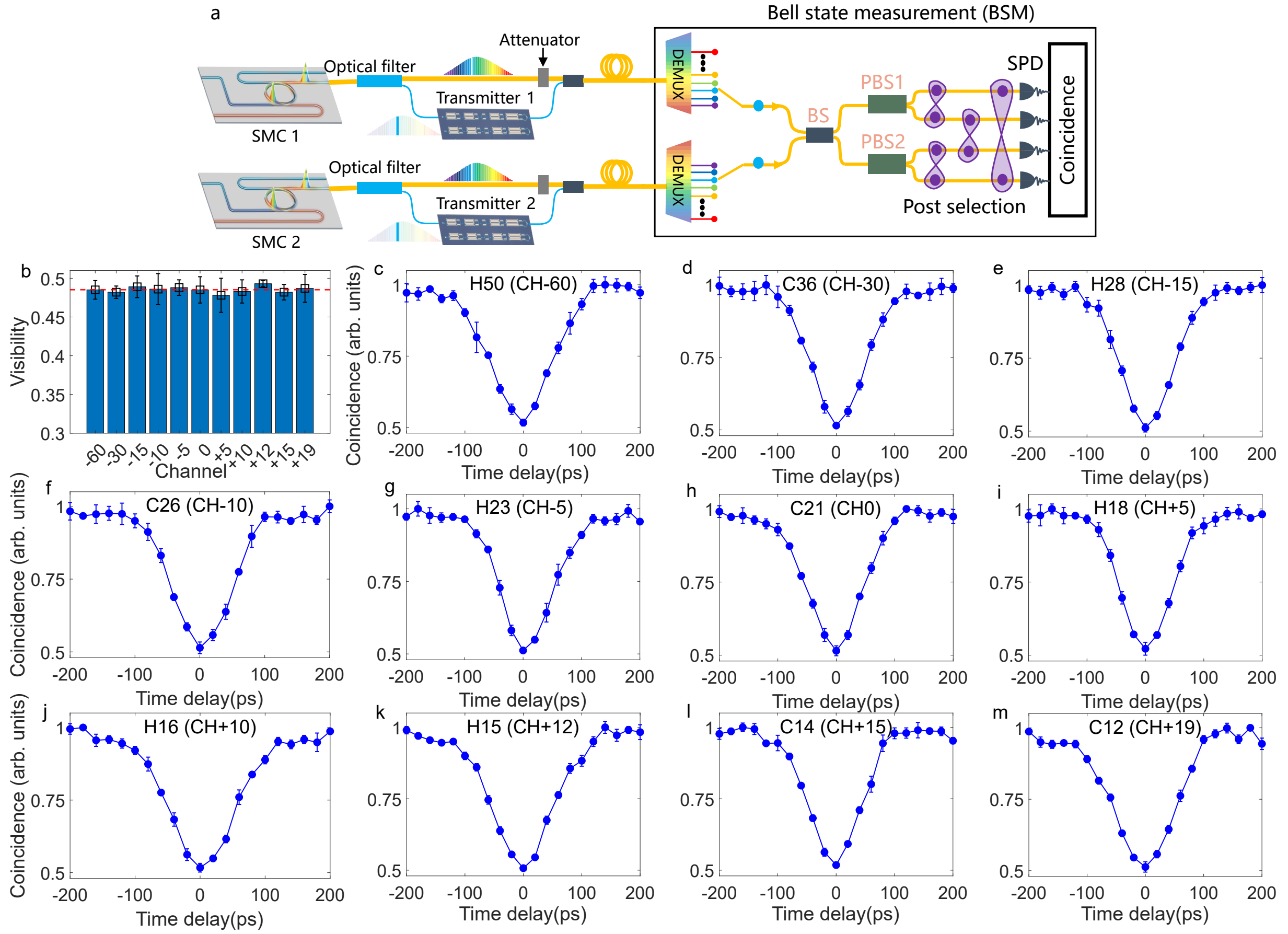

Supplementary Figure 10: **HOM interference of different frequency comb-tooth pairs. a**, Experimental setup for the HOM interference measurement. **b**, HOM interference visibilities and (**c-m**) HOM dips as a function of time delay for different comb-tooth pairs (CH-60&CH-60 to CH+19&CH+19).

equivalent-loss channel are given in Supplementary Tables 2 and 3, respectively. As the TEC temperature control for a transmitter chip integrating all encoding elements is much more stable than that for a series of bulk modulators and interferometers, the peak-to-peak intensity fluctuation is about 1.48 dB. We then implement the real-time intensity feedback using the on-chip VOA. The intensity fluctuation is finally suppressed to about 0.06 dB during long-term operation, as shown in Supplementary Fig 11. The intensity fluctuation of decoy states is at the level of 1% and ensures the practical security of the MDI quantum network. As given in Supplementary Table 2, the decoy-state intensities are 0.2097 ± 0.0012, 0.1691 ± 0.0010, and 0.0563 ± 0.0004, respectively, during 3 hours of continuous operation over a 200-km fibre. The excellent long-term intensity stability of the on-chip transmitter ensures the practical security of the MDI quantum network.

Supplementary Table 1: Detailed experimental results of the microcomb-based MDI-QKD for multiple channels over 200 km.

Channel (ITU)	H50	C36	H28	C26	H23	C21	H18	H16	C14
Q_{ss}	756234	749655	754183	751917	755020	747856	754940	755306	746848
$Q_{\mu\mu}$	75370	74600	74305	73891	73290	72079	72983	73740	70754
$Q_{\nu\nu}$	78441	79188	77436	77409	78126	75814	79278	78409	74666
$Q_{\mu\omega}$	6937	6887	6851	7022	6787	6725	6673	6893	6609
$Q_{\omega\mu}$	7539	7127	6835	7100	6914	6871	6787	6948	6772
$Q_{\nu\omega}$	2269	2315	2294	2273	2368	2338	2426	2351	2279
$Q_{\omega\nu}$	2491	2403	2350	2300	2282	2175	2324	2258	2298
$Q_{\omega\omega}$	1	0	0	0	0	0	1	0	0
E_{ss}	13036	11711	11374	12782	14916	14792	12388	12476	13083
$E_{\nu\nu}$	21904	21638	20920	21227	21206	20750	21810	21345	20835
$E_{\nu\omega}$	1142	1180	1151	1142	1169	1145	1218	1127	1145
$E_{\omega\nu}$	1251	1217	1131	1164	1112	1065	1185	1080	1121
$E_{\omega\omega}$	1	0	0	0	0	0	1	0	0
$y_{11,Z}^{L}$	1.47×10^{-6}	1.46×10^{-6}	1.47×10^{-6}	1.47×10^{-6}	1.47×10^{-6}	1.46×10^{-6}	1.47×10^{-6}	1.47×10^{-6}	1.46×10^{-6}
$e_{11}^{p,U}$	0.198	0.198	0.196	0.202	0.196	0.202	0.196	0.199	0.198
SKR per pulse	2.14×10^{-8}	2.89×10^{-8}	2.86×10^{-8}	2.43×10^{-8}	2.07×10^{-8}	1.68×10^{-8}	2.26×10^{-8}	2.67×10^{-8}	1.78×10^{-8}
SKR(bps)	53.41	72.27	71.41	60.66	51.65	42.07	56.53	66.64	44.42

Supplementary Table 2: Detailed experimental parameters and results of long-term MDI-QKD operation for channel H28 (ITU) over 200 km.

Time(s)	1000	2000	3000	4000	5000	6000	7000	8000	9000	10000
s	0.2097±0.0012									
μ	0.1691±0.0010									
ν	0.0563±0.0004									
ω	0									
Q_{ss}	763702	758725	762775	759618	759484	760120	761592	758279	760002	761597
$Q_{\mu\mu}$	72854	72571	72852	72822	72669	72657	72922	72468	72249	72964
$Q_{\nu\nu}$	76581	77093	78143	77726	78453	77623	78096	77994	77299	77720
$Q_{\mu\omega}$	6824	6745	6773	6825	6682	6870	6675	6739	6617	6695
$Q_{\omega\mu}$	6838	6556	6782	6705	6604	6712	6834	6702	6813	6758
$Q_{\nu\omega}$	2272	2393	2394	2347	2317	2307	2283	2380	2319	2349
$Q_{\omega\nu}$	2251	2276	2257	2378	2289	2288	2317	2265	2286	2254
$Q_{\omega\omega}$	0	1	0	0	0	0	0	0	0	
E_{ss}	14112	13826	13385	13421	13734	13738	13796	13395	13479	13855
$E_{\nu\nu}$	21175	21224	21359	21276	21289	21233	21367	21199	21196	21405
$E_{\nu\omega}$	1152	1208	1162	1167	1144	1138	1140	1185	1163	1217
$E_{\omega\nu}$	1124	1136	1104	1172	1132	1163	1183	1142	1109	1110
$E_{\omega\omega}$	0	0	0	0	0	0	0	0	0	
$y_{11,Z}^{L}$	1.49×10^{-6}	1.48×10^{-6}	1.49×10^{-6}	1.48×10^{-6}	1.48×10^{-6}	1.48×10^{-6}	1.48×10^{-6}	1.48×10^{-6}	1.48×10^{-6}	1.48×10^{-6}
$e_{11}^{p,U}$	0.201	0.196	0.199	0.197	0.198	0.201	0.202	0.196	0.202	0.198
SKR(pulse^{-1})	1.62×10^{-8}	1.79×10^{-8}	2.43×10^{-8}	2.28×10^{-8}	2.50×10^{-8}	2.26×10^{-8}	2.24×10^{-8}	2.57×10^{-8}	2.20×10^{-8}	1.92×10^{-8}
SKR(bps)	40.41	44.80	60.78	57.00	62.38	56.43	56.05	64.32	55.04	48.01

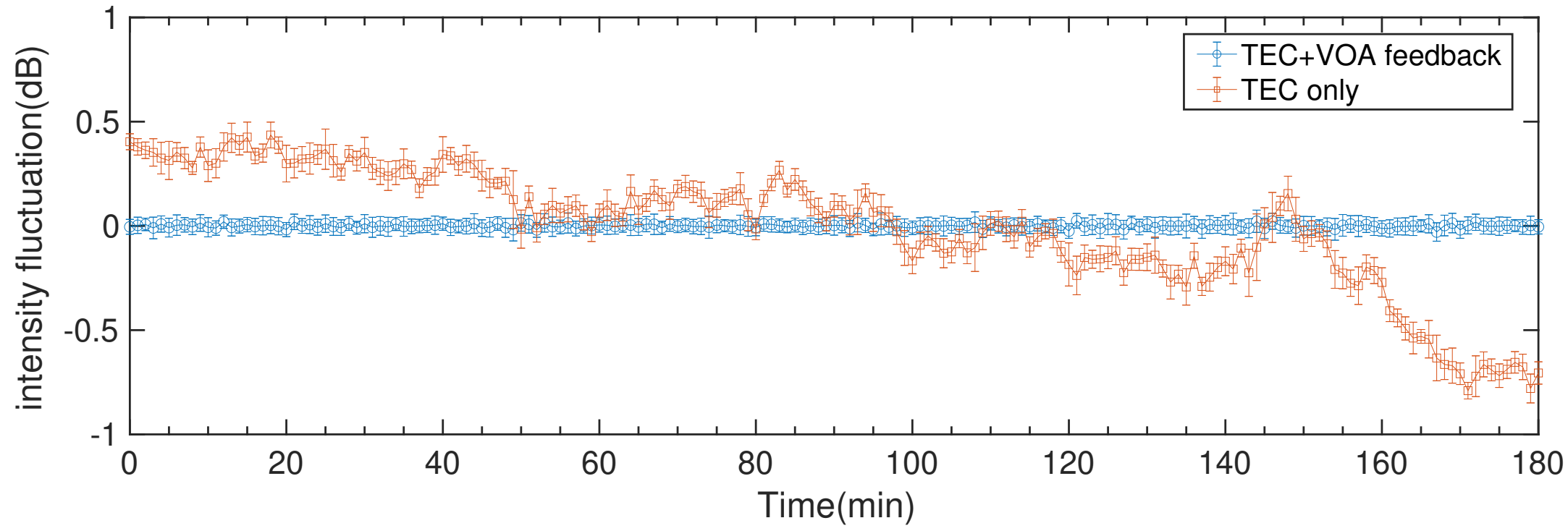

Supplementary Figure 11: **Intensity fluctuations of the transmitter chip.**

Supplementary Table 3: Detailed experimental parameters and results of long-term MDI-QKD operation for channel H28 (ITU) over a 36-dB equivalent loss.

Time(s)	1000	2000	3000	4000	5000	6000	7000	8000	9000	10000
s					0.2095±0.0031					
μ					0.1688±0.0026					
ν					0.0564±0.0009					
ω					0					
Q_{ss}	758151	759518	751536	755724	755532	761229	755972	756032	754944	756853
$Q_{\mu\mu}$	72639	72577	71554	72464	72064	72701	72068	72208	72443	72427
$Q_{\nu\nu}$	77525	77620	77452	78863	77785	78223	77962	78425	77804	78027
$Q_{\mu\omega}$	6699	6874	6936	6760	6828	6941	6648	6882	6924	6776
$Q_{\omega\mu}$	6683	6679	6558	6725	6798	6602	6671	6954	6858	6811
$Q_{\nu\omega}$	2256	2357	2386	2375	2350	2320	2256	2209	2336	2295
$Q_{\omega\nu}$	2334	2264	2315	2400	2236	2363	2368	2327	2460	2294
$Q_{\omega\omega}$	0	0	0	1	1	0	0	0	0	
E_{ss}	12432	12591	11892	11595	11263	11949	11799	11574	10941	11078
$E_{\nu\nu}$	20951	21179	21612	21319	20965	21302	21024	21074	21004	21005
$E_{\nu\omega}$	1098	1167	1199	1179	1178	1189	1108	1146	1205	1129
$E_{\omega\nu}$	1166	1098	1189	1176	1133	1191	1146	1129	1196	1115
$E_{\omega\omega}$	0	0	0	0	0	0	0	0	0	
$y_{11,Z}^{L}$	1.48×10^{-6}	1.48×10^{-6}	1.47×10^{-6}	1.47×10^{-6}	1.47×10^{-6}	1.48×10^{-6}	1.47×10^{-6}	1.47×10^{-6}	1.47×10^{-6}	1.47×10^{-6}
$e_{11}^{p,U}$	0.197	0.200	0.197	0.191	0.194	0.197	0.193	0.192	0.185	0.193
SKR(pulse^{-1})	2.65×10^{-8}	2.50×10^{-8}	2.33×10^{-8}	3.32×10^{-8}	3.40×10^{-8}	2.84×10^{-8}	3.10×10^{-8}	3.64×10^{-8}	3.47×10^{-8}	3.43×10^{-8}
SKR(bps)	66.16	62.57	58.21	82.98	84.93	70.97	77.59	91.07	86.76	85.65

Supplementary Note 8 – Potential Time-Dependent-Phase Side Channel

For high-speed QKD, the time-dependent-phase side channel may affect the secure key rate when the optical intensity profile of the encoded photons is relatively wide compared to the bandwidth of the driven signals loaded on phase modulators. We evaluate the hidden multidimensional modulation side channels following Ref. (*17*). Considering the linear independence of bases in higher dimensions, only the component of the high-dimensional modulation lying outside the encoding space contributes to Eve's unambiguous state discrimination (USD) measurement (*18*). Hence, the

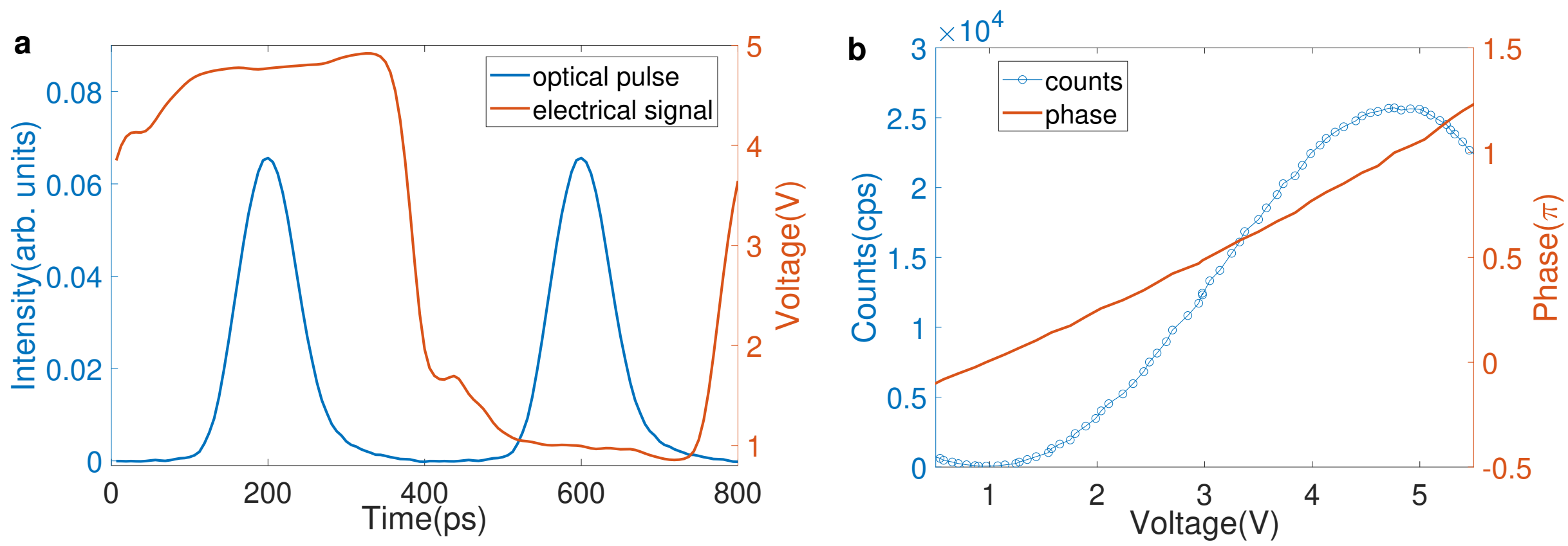

Supplementary Figure 12: **High-speed drive signal and phase modulation of optical pulses. a**, Optical pulses and electrical drive signal at a 2.5 GHz baud rate. **b**, Modulation phase and corresponding count rate versus drive voltage.

imperfect state in dimension $|1\rangle$ can be expressed in a more general form

$$|1'\rangle = \sqrt{1-\epsilon}|1\rangle + \sqrt{\epsilon_Q}|0\rangle + \sqrt{\epsilon_S}|2\rangle \tag{35}$$

where $\epsilon = 1 - |\langle\theta|\psi\rangle|^2 = \epsilon_Q + \epsilon_S$ is the preparation error, $|\psi\rangle = \int_{-\infty}^{\infty} dt\sqrt{f(t)}|t\rangle \otimes \frac{|H\rangle + e^{i\psi(t)}|V\rangle}{\sqrt{2}}$ is the imperfect preparation state in the high dimension, $|\theta\rangle = \int_{-\infty}^{\infty} dt\sqrt{f(t)}|t\rangle \otimes \frac{|H\rangle + e^{i\theta}|V\rangle}{\sqrt{2}}$ is the closest state to ψ in the encoding space and and $\theta = \int_{-\infty}^{\infty} dt f(t)\psi(t)$. The Qubit flaw is $|0\rangle = \int_{-\infty}^{\infty} dt\sqrt{f(t)}|t\rangle \otimes \frac{|H\rangle - e^{i\theta}|V\rangle}{\sqrt{2}}$ and $\epsilon_Q = |\langle 0|\psi\rangle|^2$. The qubit flaw term $\epsilon_Q|0\rangle$ leads to a measurable quantum bit error rate (QBER) and has little contribution to the high-dimension side channel, while the term $\sqrt{\epsilon_S}|2\rangle$, which lies in the higher dimension beyond the encoding space, should be evaluated carefully. According to the above analysis, we obtain

$$\epsilon_Q = \frac{1-|\xi|}{2}, \epsilon_S = \frac{1-|\xi|^2}{4} = \epsilon_Q - {\epsilon_Q}^2 \tag{36}$$

where $\xi = \int_{-\infty}^{\infty} dt f(t) e^{i\psi(t)}$. See Refs. (*17, 18*) for more details of the side-channel analysis.

To evaluate the amplitude in the third dimension of the imperfect state, we first measure the normalized temporal intensity profile of the optical pulse and the electrical drive signal in Supplementary Fig. 12a. Since the voltage-dependent transmittance curve of the modulator has been precisely pre-calibrated, we map the temporal drive signal to the phase shift according to the voltage-dependent transmittance curve in Supplementary Fig. 12b. The high and low voltage levels of the

400-ps-width square wave correspond to phase shifts of 0 ($|H\rangle$) and π ($|V\rangle$), respectively. The calculated $\epsilon_S = 1.2 \times 10^{-4}$ is at the same level of as the 12.5-ps pulse case given in Ref. (*17*). This would lead to a very slight decrease in SKR within the linear region and would guarantee the practical security of the MDI quantum network.

The Trojan Horse attack is another common attack against practical QKD systems, especially plug-and-play systems (*19–21*). For a one-way MDI system, the security loopholes exist only in the source and the Trojan Horse attack can be effectively defended against by the isolators at the output port of the transmitter. Moreover, benefiting from the integrated microstructures (*22*) as well as various readily implementable defense elements (*23, 24*), chip-based transmitters are more robust against potential attacks.

References

1. T. Herr, *et al.*, Temporal solitons in optical microresonators. *Nature Photonics* **8** (2), 145–152 (2014).

2. M. Qu, *et al.*, Towards a compact soliton microcomb fully referenced on atomic reference. *Optics Express* **32** (20), 34948 (2024), doi:10.1364/OE.533707.

3. Y.-H. Zhou, Z.-W. Yu, X.-B. Wang, Making the decoy-state measurement-device-independent quantum key distribution practically useful. *Physical Review A* **93** (4), 042324 (2016).

4. F.-Y. Lu, *et al.*, Efficient decoy states for the reference-frame-independent measurement-device-independent quantum key distribution. *Physical Review A* **101** (5), 052318 (2020).

5. H. Chernoff, A measure of asymptotic efficiency for tests of a hypothesis based on the sum of observations. *The Annals of Mathematical Statistics* **23** (4), 493–507 (1952).

6. C. Jiang, Z.-W. Yu, X.-B. Wang, Measurement-device-independent quantum key distribution with source state errors and statistical fluctuation. *Physical Review A* **95** (3), 032325 (2017).

7. C. Jiang, Z.-W. Yu, X.-L. Hu, X.-B. Wang, Higher key rate of measurement-device-independent quantum key distribution through joint data processing. *Physical Review A* **103** (1), 012402 (2021).

8. Z.-W. Yu, Y.-H. Zhou, X.-B. Wang, Statistical fluctuation analysis for measurement-device-independent quantum key distribution with three-intensity decoy-state method. *Physical Review A* **91** (3), 032318 (2015).

9. M. Hayashi, T. Tsurumaru, Concise and tight security analysis of the Bennett-Brassard 1984 protocol with finite key lengths. *New Journal of Physics* **14**, 093014 (2012).

10. M. Tomamichel, C. C. W. Lim, N. Gisin, R. Renner, Tight finite-key analysis for quantum cryptography. *Nature Communications* **3**, 634 (2012).

11. M. Curty, *et al.*, Finite-key analysis for measurement-device-independent quantum key distribution. *Nature communications* **5**, 3732 (2014).

12. M. A. Vorontsov, V. P. Sivokon, Stochastic parallel-gradient-descent technique for high-resolution wave-front phase-distortion correction. *Journal of the Optical Society of America A* **15** (10), 2745 (1998).

13. W. Li, *et al.*, High-rate quantum key distribution exceeding 110 Mb s^{1}. *Nature Photonics* **17** (5), 416–421 (2023).

14. H.-L. Yin, *et al.*, Measurement-Device-Independent Quantum Key Distribution Over a 404 km Optical Fiber. *Physical Review Letters* **117** (19), 190501 (2016).

15. K. Wei, *et al.*, High-Speed Measurement-Device-Independent Quantum Key Distribution with Integrated Silicon Photonics. *Physical Review X* **10** (3), 031030 (2020).

16. C. B. Czegledi, M. Karlsson, E. Agrell, P. Johannisson, Polarization Drift Channel Model for Coherent Fibre-Optic Systems. *Scientiic Reports* **6** (1), 21217 (2016).

17. A. Gnanapandithan, L. Qian, H.-K. Lo, Hidden Multidimensional Modulation Side Channels in Quantum Protocols. *Physical Review Letters* **134** (13), 130802 (2025), doi:10.1103/PhysRevLett.134.130802.

18. G. Currás-lorenzo, M. Pereira, M. Curty, K. Tamaki, Security framework for quantum key distribution with imperfect sources. *Optica Quantum* **3** (6), 525 (2025), doi:10.1364/OPTICAQ.569424.

19. N. Gisin, S. Fasel, B. Kraus, H. Zbinden, G. Ribordy, Trojan-horse attacks on quantum-key-distribution systems. *Physical Review A* **73** (2), 022320 (2006), doi:10.1103/PhysRevA.73.022320.

20. C.-H. F. Fung, B. Qi, K. Tamaki, H.-K. Lo, Phase-remapping attack in practical quantum-key-distribution systems. *Physical Review A* **75** (3), 032314 (2007), doi:10.1103/PhysRevA.75.032314.

21. N. Jain, *et al.*, Risk Analysis of Trojan-Horse Attacks on Practical Quantum Key Distribution Systems. *IEEE Journal of Selected Topics in Quantum Electronics* **21** (3), 6600710 (2015), doi:10.1109/JSTQE.2014.2365585.

22. H. Tan, W. Li, L. Zhang, K. Wei, F. Xu, Chip-Based Quantum Key Distribution against Trojan-Horse Attack. *Physical Review Applied* **15** (6), 064038 (2021).

23. J. Lapointe, C. Coia, A. Dupont, R. Vallée, Passive broadband Faraday isolator for hybrid integration to photonic circuits without lens and external magnet. *Nature Photonics* **19** (March), 248–257 (2025), doi:10.1038/s41566-024-01601-0.

24. M. Chen, *et al.*, Optical fuse based on the photorefractive effect for defending the light-injection attacks of quantum key distribution. *arXiv2512.10205* (2025), `http://arxiv.org/abs/2512.10205`.
